# Supplementary material for: The splicing regulators TIA1 and TIAL1 are required for the expression of the DNA damage repair machinery during B cell lymphopoiesis
Source: Cell Rep. 2022 Dec 20;41(12):111869. doi: 10.1016/j.celrep.2022.111869 (PMC9794549; doi:10.1016/j.celrep.2022.111869)
Supplement: Document S1. Figures S1–S6 [file mmc1.pdf]

Cell Reports, Volume *41*

Supplemental information

The splicing regulators TIA1 and TIAL1 are required for the expression of the DNA damage repair machinery during B cell lymphopoiesis

Ines C. Osma-Garcia, Dunja Capitan-Sobrino, Mailys Mouysset, Yann Aubert, Orlane Maloudi, Martin Turner, and Manuel D. Diaz-Muñoz

Suppl. Figure 1

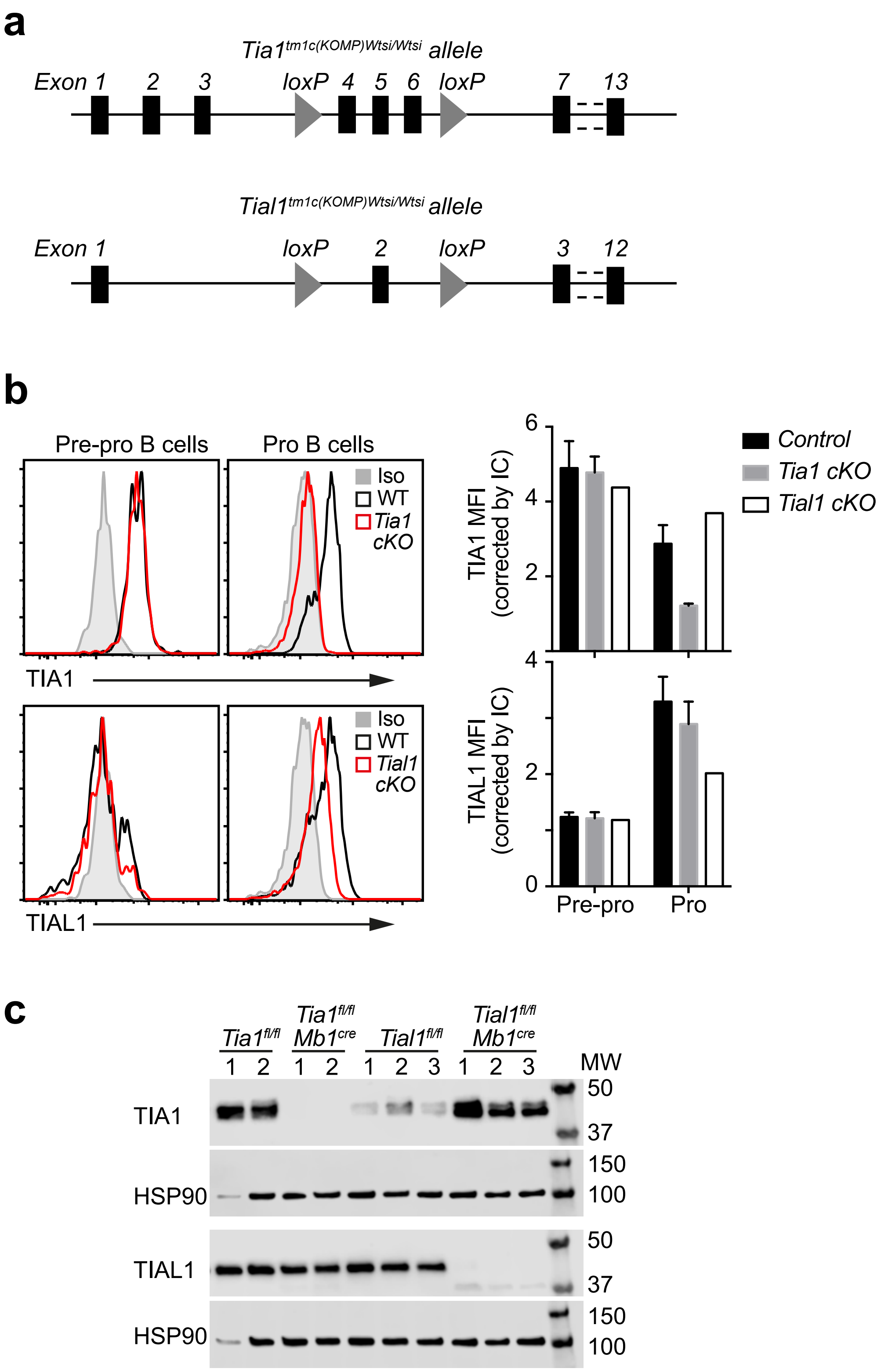

Figure S1. Targeting strategy for conditional deletion of *Tia1* and *Tial1*. Related to Figure 1.

(A) Schematic representation of the *Tia1*$^{tm1c(KOMP)Wtsi/Wtsi}$ and *Tial1*$^{tm1c(KOMP)Wtsi/Wtsi}$ alleles (*Tia1*$^{fl/fl}$ and *Tial1*$^{fl/fl}$) indicating the exons flanked by loxP sites for conditional Mb1-Cre mediated recombination.

(B) Analysis by flow cytometry of *Tia1* and *Tial1* conditional deletion in pro-B cells. Left panels, representative flow cytometry histograms showing the expression of TIA1 and TIAL1 in pre-pro B cells and pro-B cells. Right panels, quantitation of TIA1 and TIAL1 expression (mean $\pm$ SD, n=3 mice/genotype).

(C) Immunoblot of TIA1 and TIAL1 in isolated CD19^{+} splenic B cells from control or single *Tia1* cKO or single *Tial1* cKO mice. HSP90 was used as loading control. Data from n=2-3 mice/genotype.

Figure S2

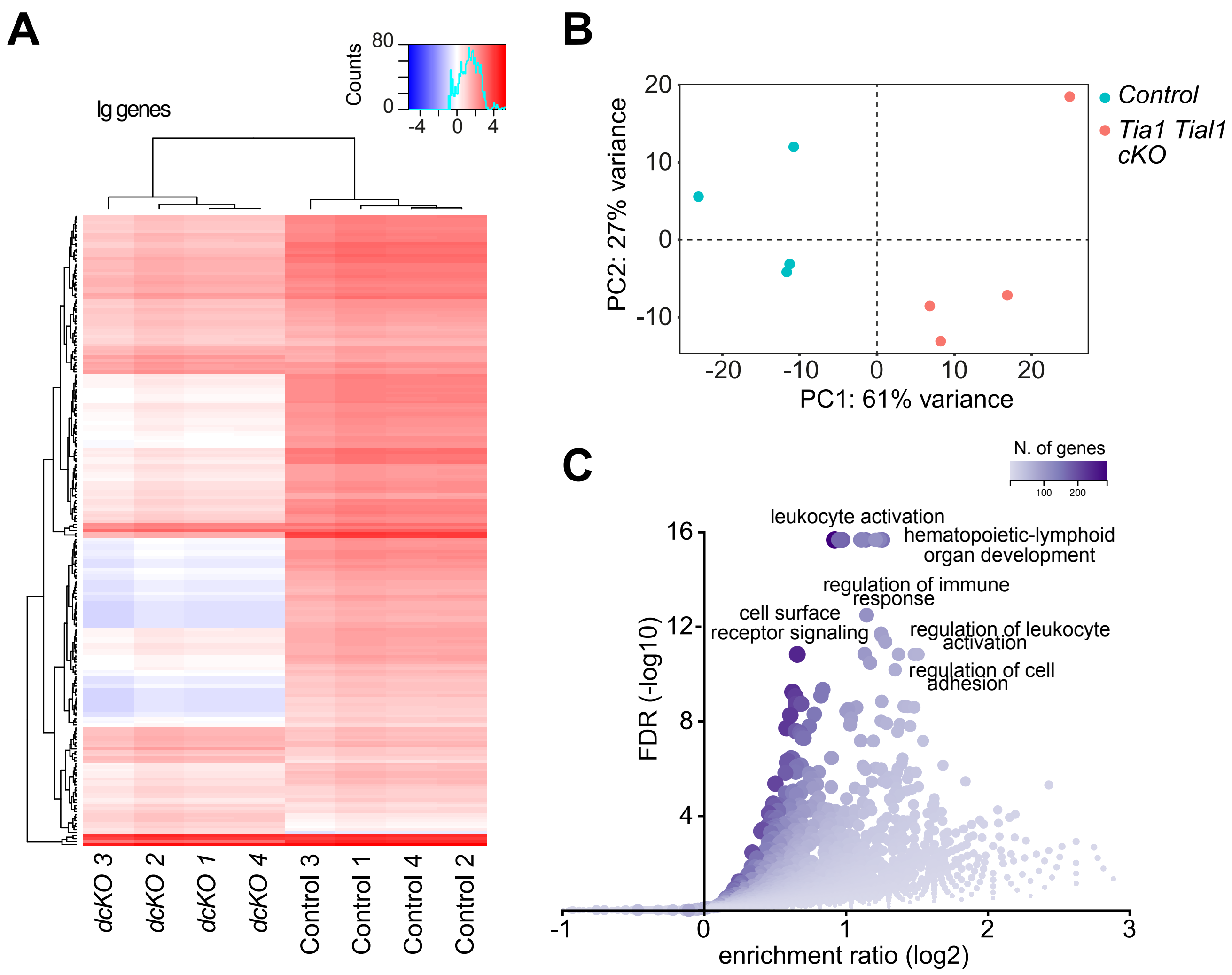
A
Ig genes
Counts
80
0
-4
0
4
dcKO 3
dcKO 2
dcKO 1
dcKO 4
Control 3
Control 1
Control 4
Control 2
B
PC2: 27% variance
PC1: 61% variance
Control
Tia1 Tial1 cKO
C
N. of genes
100
200
FDR (-log10)
enrichment ratio (log2)
leukocyte activation
hematopoietic-lymphoid organ development
regulation of immune response
cell surface receptor signaling
regulation of leukocyte activation
regulation of cell adhesion

Figure S2. Transcriptomics analysis of double *Tia1* and *Tial1* cKO pro-B cells. Related to Figure 4.

(A) Heatmap showing the expression of Ig genes in pro-B cells from control and double *Tia1* and *Tial1* cKO mice quantified by RNAseq (n=4 samples per genotype, DESeq2 analysis).

(B) Principal component analysis of transcriptomic variance in pro-B cells from control and double *Tia1* and *Tial1* cKO mice.

(C) Dot plot showing the cellular pathways that are enriched with genes differentially expressed in double *Tia1* and *Tial1 cKO* pro-B cells (GSEA performed with WebGestalt using GO terms linked to biological processes).

Figure S3

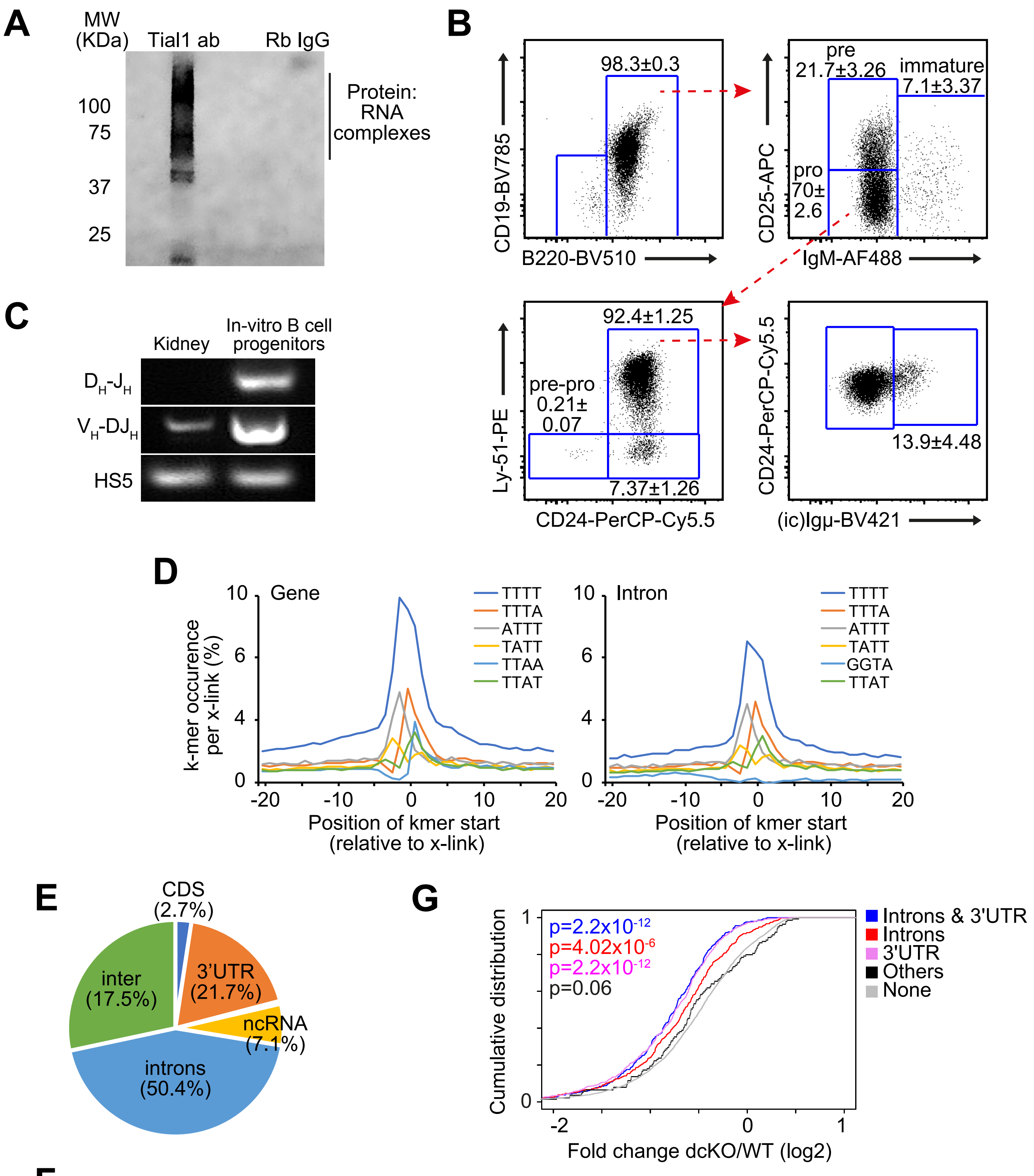

F

Xlink found in	Total genes	Genes with high confident xlink site (FDR<0.05)
Intron only	7272	913
3'UTR only	1361	1960
Intron & 3'UTR	6945	915
Others	2068	338

Figure S3. TIAL1:RNA interactome analysis in pro-B cells. Related to Figure 4.

(A) Detection of TIAL1:RNA complexes immunoprecipitated for iCLIP library construction.

(B) Characterization by flow cytometry of progenitor B cells expanded in-vitro and used for TIAL1:RNA interactome analyses.

(C) PCR analysis of VDJ recombination in *in-vitro* cultured progenitor B cells. D_H-J_H and V_H-DJ_H events were assessed after isolation of genomic DNA. HS5 sequence was used as control. Genomic DNA from the kidney of a C57BL/6 mouse was used as negative control.

(D) TIAL1 binding motif analysis. The frequency of 4-mers was quantify relative to TIAL1 cross(x)-link sites annotated to gene or intronic sequences. Only top 6 k-mer occurrences are shown.

(E) Percentage of TIAL1 crosslink sites annotated to introns, coding sequences (CDS), 3'UTRs, non-coding RNAs (ncRNAs) or intergenic sequences.

(F) Summary of target genes classified based on TIAL1 binding only in introns or 3'UTRs, or in both. The number of genes with high confident cross(x)-link sites is indicated (FDR<0.05).

(G) Cumulative distribution analysis of global changes in gene expression in double *Tia1* and *Tial1* cKO pro-B cells compared to control pro-B cells. Genes were sorted based on the detection of TIAL1 crosslink peak sites (FDR<0.05) in introns, 3'UTR and others. Kolmogorov-Smirnov tests were performed comparing a TIAL1 targeted gene group against the group of genes non-targeted by TIAL1.

Figure S4

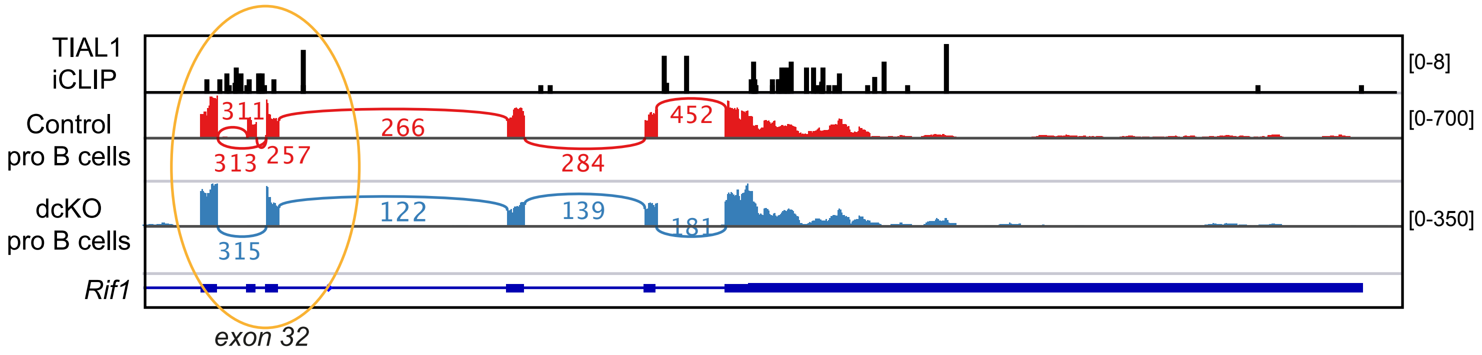

TIAL1
iCLIP
[0-8]
Control
pro B cells
311
266
452
313
257
284
[0-700]
dcKO
pro B cells
122
139
181
315
[0-350]
Rif1
exon 32

Figure S4. TIA1 and TIAL1 control *Rif1* mRNA splicing and expression. Related to Figure 5.

Sashimi plot showing alternative splicing of exon 32 of *Rif1* in pro-B cells from double *Tia1* and *Tial1* cKO mice. The number of reads annotated to the exon-exon junctions and TIAL1 crosslink sites are shown. Scales indicate the number of unique cDNA counts annotated per TIAL1 crosslink site or exon abundance.

Figure S5

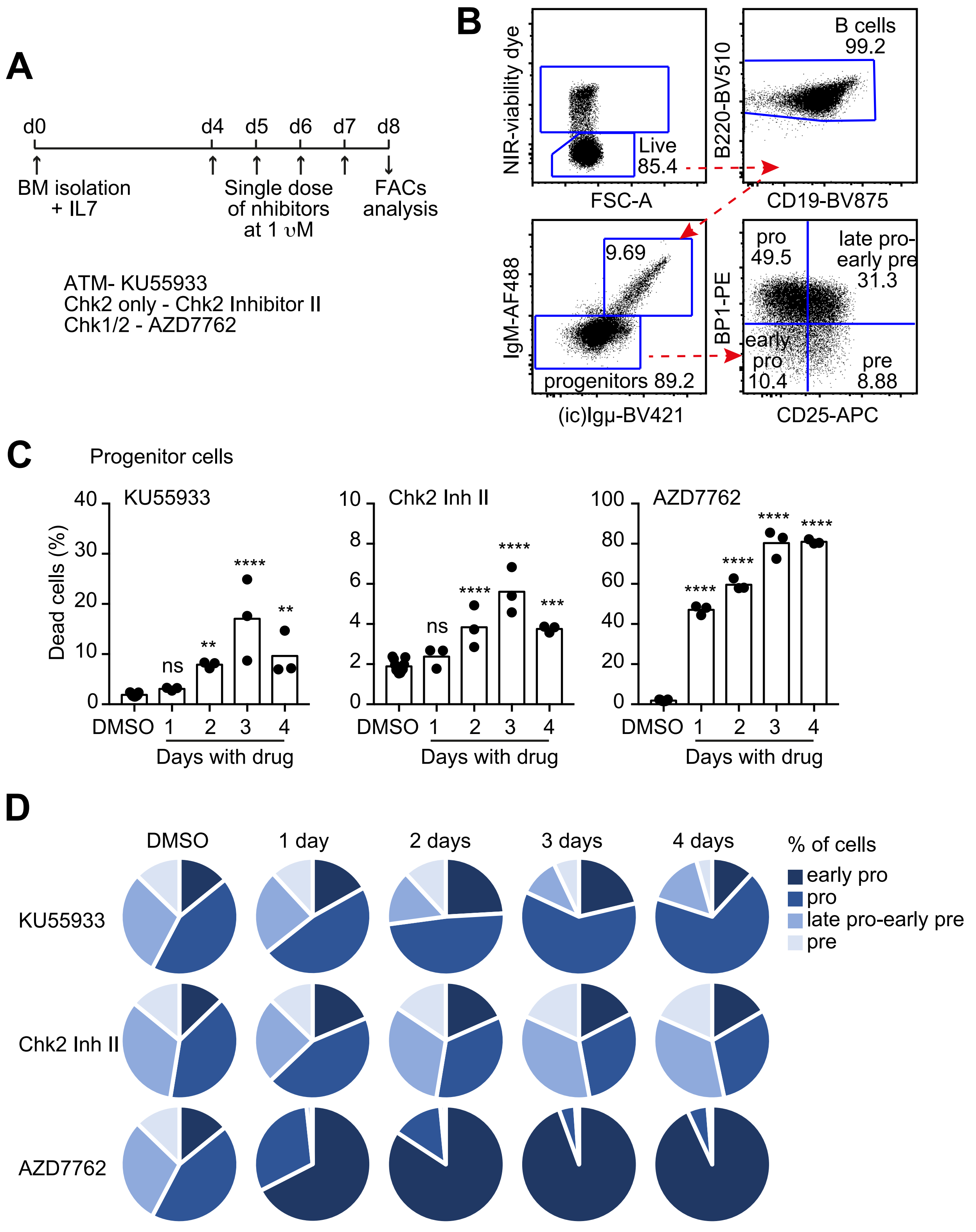
A
d0
d4
d5
d6
d7
d8
BM isolation
+ IL7
Single dose
of nhibitors
at 1 υM
FACs
analysis
ATM- KU55933
Chk2 only - Chk2 Inhibitor II
Chk1/2 - AZD7762
B
NIR-viability dye
FSC-A
Live
85.4
B220-BV510
CD19-BV875
B cells
99.2
IgM-AF488
(ic)Igμ-BV421
9.69
progenitors 89.2
BP1-PE
CD25-APC
pro
49.5
late pro-
early pre
31.3
early
pro
10.4
pre
8.88
C
Progenitor cells
Dead cells (%)
KU55933
Chk2 Inh II
AZD7762
ns
**
****
***
DMSO 1 2 3 4
Days with drug
D
DMSO
1 day
2 days
3 days
4 days
KU55933
Chk2 Inh II
AZD7762
% of cells
early pro
pro
late pro-early pre
pre

Figure S5. TIA1 and TIAL1 control *Rif1* mRNA splicing and expression. Related to Figure 6.

(A) Experimental set up to analyse the impact of ATM and CHK inhibitors in progenitor B cells *in-vitro*. Inhibitors were added into the cell culture once at the indicated time point.

(B) FACs gating strategy to assess the different subsets of progenitor B cells.

(C) Analysis of cell death in B cell cultures treated with ATM and CHK inhibitors.

(D) Pie charts showing the distribution of cell population in the cell cultures analysed in B. Data is representative from two independent experiments. In each experiment, bone marrow B cells were isolated from three mice and cultured independently to assess for biological variability. Mann-Whitney tests were performed for statistical analysis.

Figure S6

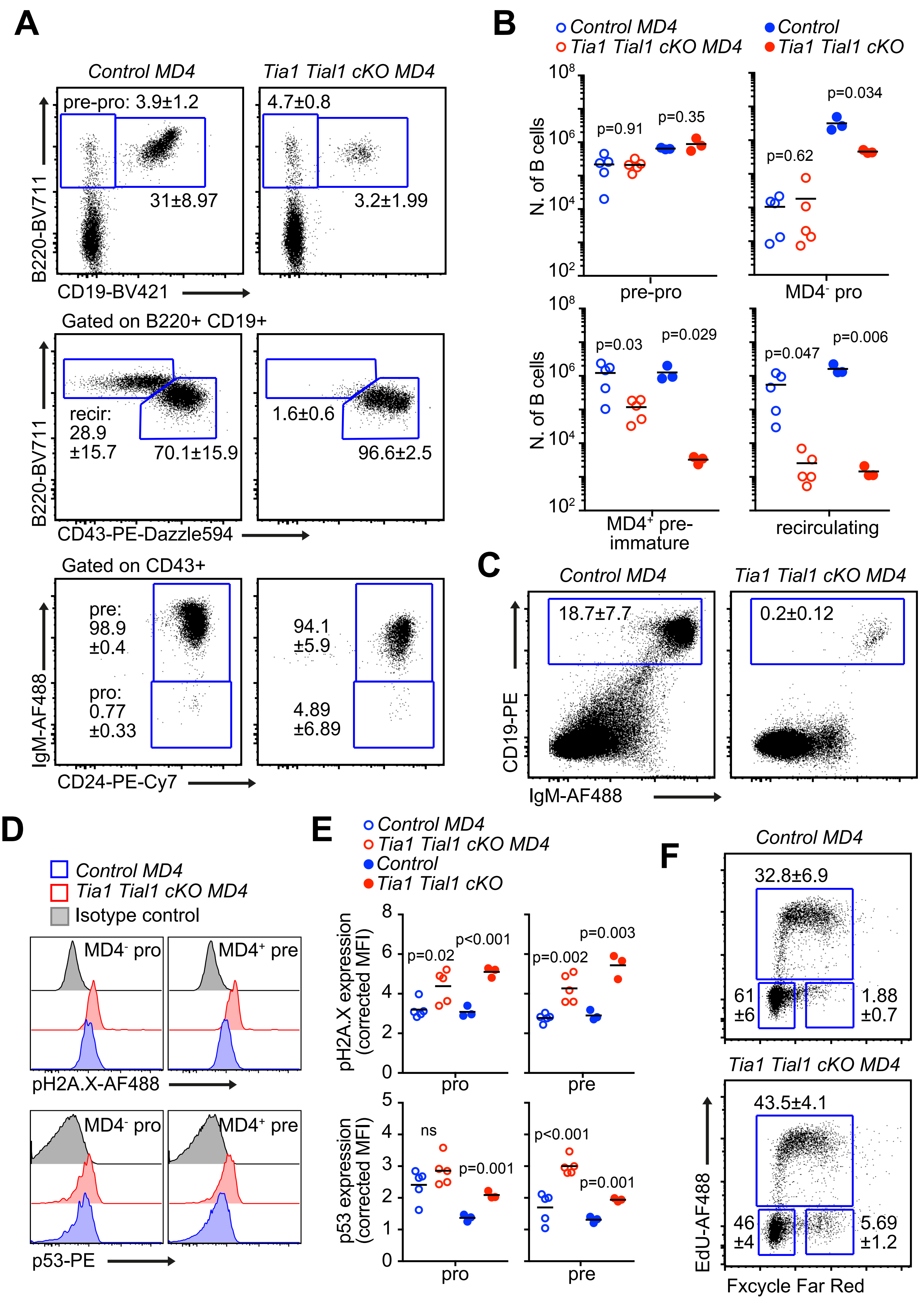
A
Control MD4
Tia1 Tial1 cKO MD4
pre-pro: 3.9±1.2
31±8.97
4.7±0.8
3.2±1.99
B220-BV711
CD19-BV421
Gated on B220+ CD19+
recir: 28.9 ±15.7
70.1±15.9
1.6±0.6
96.6±2.5
CD43-PE-Dazzle594
Gated on CD43+
pre: 98.9 ±0.4
pro: 0.77 ±0.33
94.1 ±5.9
4.89 ±6.89
IgM-AF488
CD24-PE-Cy7
B
Control MD4
Tia1 Tial1 cKO MD4
Control
Tia1 Tial1 cKO
N. of B cells
p=0.91
p=0.35
pre-pro
p=0.62
p=0.034
MD4- pro
p=0.03
p=0.029
MD4+ pre-immature
p=0.047
p=0.006
recirculating
C
Control MD4
Tia1 Tial1 cKO MD4
18.7±7.7
0.2±0.12
CD19-PE
IgM-AF488
D
Control MD4
Tia1 Tial1 cKO MD4
Isotype control
MD4- pro
MD4+ pre
pH2A.X-AF488
p53-PE
E
Control MD4
Tia1 Tial1 cKO MD4
Control
Tia1 Tial1 cKO
pH2A.X expression (corrected MFI)
p=0.02
p<0.001
p=0.002
p=0.003
pro
pre
p53 expression (corrected MFI)
ns
p=0.001
p<0.001
p=0.001
F
Control MD4
32.8±6.9
61 ±6
1.88 ±0.7
Tia1 Tial1 cKO MD4
43.5±4.1
46 ±4
5.69 ±1.2
EdU-AF488
Fxcycle Far Red

Figure S6. Expression of a BCR transgene fails to rescue *Tia1* and *Tial1* double KO progenitor B cells from exacerbated DNA damage. Related to Figure 7.

(A) Flow cytometry analysis of B cell subpopulations in the BM of *Rag2*$^{-/-}$ mice reconstituted with BM cells from *Tia1*$^{fl/fl}$ *Tial1*$^{fl/fl}$ MD4-tg (control MD4) mice or *Tia1*$^{fl/fl}$ *Tial1*$^{fl/fl}$ MD4-tg *Mb1*^{Cre} (*Tia1 Tial1* cKO MD4) mice. n=5 mice per genotype. The percentage of cells in each gate is shown as the mean value ± SD.

(B) Quantitation of the number of B cell progenitors in mice shown in A.

(C) Flow cytometry dot plots showing the percentage of B cells in the spleen of mice shown in A.

(D) Histograms showing the expression of pSer139-H2A.X and p53 in B cell progenitors expressing or not the MD4 BCR transgene.

(E) Quantitation of expression of pSer139-H2A.X and p53 in pro- and pre-B cells from mice described in A. Analysis of pSer139-H2A.X and p53 in *Tia1*$^{fl/fl}$ *Tial1*$^{fl/fl}$ (control) and *Tia1*$^{fl/fl}$ *Tial1*$^{fl/fl}$ *Mb1*^{Cre} (*Tia1 Tial1* cKO) was performed in parallel.

(F) Analysis of cell cycle progression in MD4^{+} progenitor B cells in mice shown in A. Unpaired t-test were performed in statistical analyses in B and E. Each data point is from one mouse.
