# Supplementary material for: The splicing regulators TIA1 and TIAL1 are required for the expression of the DNA damage repair machinery during B cell lymphopoiesis
Source: Cell Rep. 2022 Dec 20;41(12):111869. doi: 10.1016/j.celrep.2022.111869 (PMC9794549; doi:10.1016/j.celrep.2022.111869)
Supplement: Document S2. Article plus supplemental information [file mmc7.pdf]

Cell Reports

Article

The splicing regulators TIA1 and TIAL1 are required for the expression of the DNA damage repair machinery during B cell lymphopoiesis

Graphical abstract

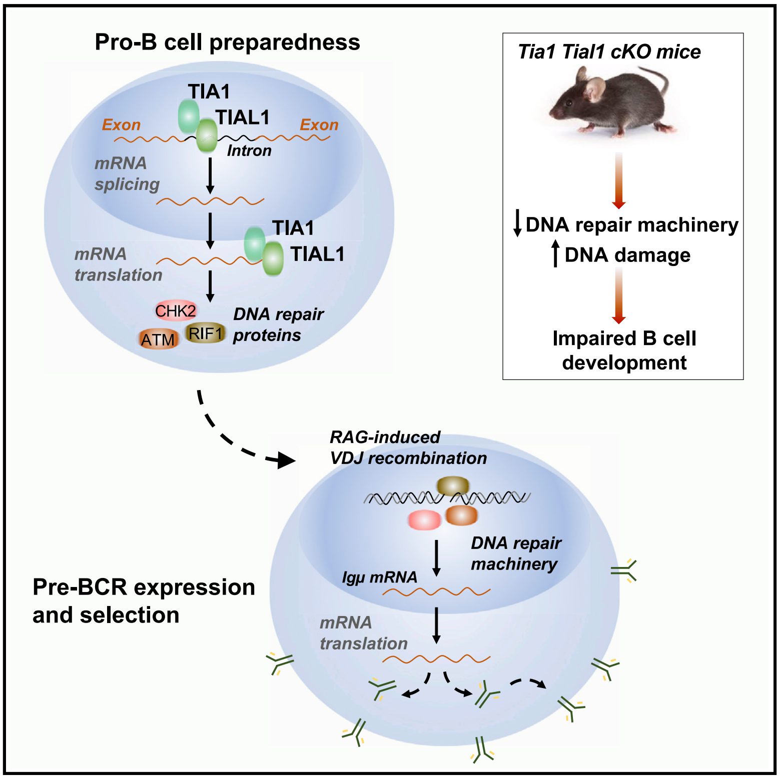

Authors

Ines C. Osma-Garcia, Dunja Capitan-Sobrino, Mailys Mouysset, Yann Aubert, Orlane Maloudi, Martin Turner, Manuel D. Diaz-Muñoz

Correspondence

manuel.diaz-munoz@inserm.fr

In brief

Tight control of VDJ recombination, cell selection, and cell proliferation is required for the development of an ample B cell repertoire. Osma-Garcia et al. report the need of TIA1/TIAL1-dependent post-transcriptional regulation for splicing and expression of key DNA damage sensors and repair proteins that enable BCR formation and B cell generation.

Highlights

- Post-transcriptional regulation by TIA1 and TIAL1 is needed for B cell development
- VDJ recombination is impaired in the absence of TIA1 and TIAL1
- TIA1 and TIAL1 control exon definition and splicing in progenitor B cells
- TIA1 and TIAL1 are required for the expression of DNA damage repair machinery

Osma-Garcia et al., 2022, Cell Reports *41*, 111869
December 20, 2022 © 2022 The Authors.
https://doi.org/10.1016/j.celrep.2022.111869

CellPress

Cell Reports

CellPress
OPEN ACCESS

Article

The splicing regulators TIA1 and TIAL1 are required for the expression of the DNA damage repair machinery during B cell lymphopoiesis

Ines C. Osma-Garcia,[1] Dunja Capitan-Sobrino,[1] Mailys Mouysset,[1] Yann Aubert,[1] Orlane Maloudi,[1] Martin Turner,[2] and Manuel D. Diaz-Muñoz[1,3,*]

[1]Toulouse Institute for Infectious and Inflammatory Diseases (INFINITy), Inserm UMR1291, CNRS UMR5051, University Paul Sabatier, CHU Purpan, Toulouse 31024, France

[2]Immunology Program, The Babraham Institute, Babraham Research Campus, Cambridge CB22 3AT, UK

[3]Lead contact

*Correspondence: manuel.diaz-munoz@inserm.fr

https://doi.org/10.1016/j.celrep.2022.111869

SUMMARY

B cell lymphopoiesis requires dynamic modulation of the B cell transcriptome for timely coordination of somatic mutagenesis and DNA repair in progenitor B (pro-B) cells. Here, we show that, in pro-B cells, the RNA-binding proteins T cell intracellular antigen 1 (TIA1) and TIA1-like protein (TIAL1) act redundantly to enable developmental progression. They are global splicing regulators that control the expression of hundreds of mRNAs, including those involved in DNA damage repair. Mechanistically, TIA1 and TIAL1 bind to 5′ splice sites for exon definition, splicing, and expression of DNA damage sensors, such as *Chek2* and *Rif1.* In their absence, pro-B cells show exacerbated DNA damage, altered P53 expression, and increased cell death. Our study uncovers the importance of tight regulation of RNA splicing by TIA1 and TIAL1 for the expression of integrative transcriptional programs that control DNA damage sensing and repair during B cell development.

INTRODUCTION

Timely control of somatic recombination and DNA damage repair is essential for the development of a pool of B lymphocytes with diverse antigen receptor specificities. After commitment of bone marrow (BM) progenitor cells into the B cell lineage, rearrangement in progenitor (pro) B cells of the variable (V_H), diversity (D_H), and joining (J_H) gene segments of the immunoglobulin heavy chain (IgH) marks a critical checkpoint leading to the expression of a pre-B cell receptor (pre-BCR) for further precursor (pre) B cell selection and expansion. Subsequent recombination of the variable (V_L) and joining (J_L) gene segments of the immunoglobulin light chain (IgL) in pre-B cells generates the BCR that drives selection and differentiation of non-autoreactive B cell clones.[1,2]

DNA damage activates multi-functional genetic programs that couple V(D)J recombination, DNA repair, cell-cycle progression and B cell selection.[3] Transcription factors, such as E2A, Foxo-1, EBF1, PAX5, and Ikaros, initiate the genetic programs for B lymphocyte commitment and DNA mutagenesis in developing B cells.[4,5] Further post-transcriptional regulation by RNA-binding proteins (RBPs) is required for translation of these transcriptional programs into protein networks for timely control of cell quiescence, somatic recombination, and selection.[6] RBPs modulate the editing, splicing, stability, and translation of newly synthesized transcripts, including those that coordinate cellular responses to DNA damage.[7–9] RBPs also direct the maintenance of genome integrity. They are recruited to the sites of DNA damage for resolution of DNA:RNA hybrids formed during RNA processing and DNA replication. In addition, they take an active role in DNA double-strand break (DSB) signaling and repair by non-homologous end joining (NHEJ) recombination and homologous recombination (HR).[10]

RBPs have other functions beyond regulation of DNA damage in developing B cells. PTBP1 and PTBP2 preserve the splicing and expression of cell-cycle regulators for the expansion of pro-B cells after functional IgH locus recombination.[11] Similarly, RNA m^{6}A methylation by METTL14 is required for IL-7-mediated proliferation of pro-B cells independently of VDJ recombination and pre-BCR expression.[12] Timely control of mRNA stability and translation is also essential during B cell development. ZFP36L1 and ZFP36L2 promote mRNA decay of cell-cycle genes enforcing pro-B cell quiescence for successful IgH locus recombination.[13] This is further supported by miRNAs and CNOT3, part of the CCR4-NOT complex, that limit the expression of pro-apoptotic genes (*Bcl2l11* and *Trp53*) in pro-B cells to allow B cell developmental transition.[14–16] CNOT3 also interacts with EBF1 for sustained transcription of *Pax5* and *Ebf1* and their associated transcriptional programs.[16] Altogether, these studies highlight an emerging picture in which post-transcriptional regulatory mechanisms control the genetic programs that enable development of B cells.

In this study, we demonstrate that the RBP T cell intracellular antigen 1 (TIA1) and its paralog TIA1-like protein (TIAL1) control the expression of the integrative DNA damage response required for mutagenesis and development of progenitor B cells. TIA1 and

© 2022 The Authors.
This is an open access article under the CC BY license (http://creativecommons.org/licenses/by/4.0/).

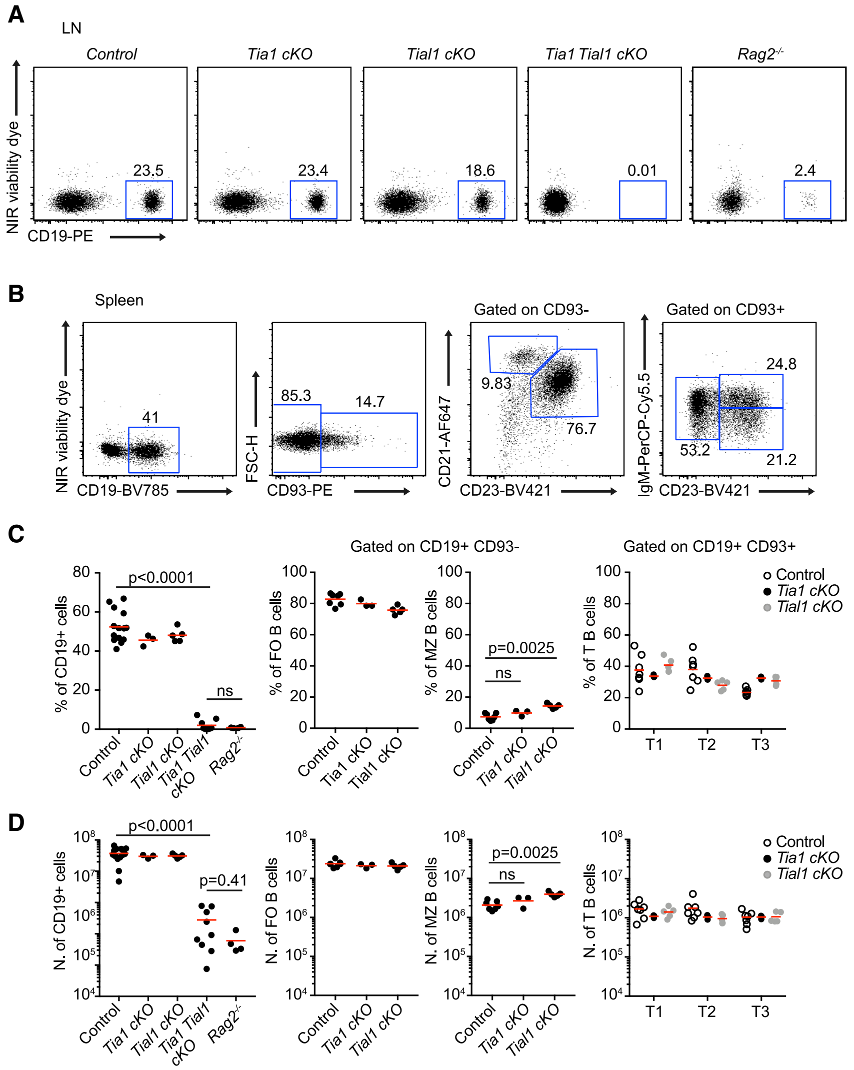

Figure 1. B cell lymphopenia in the absence of TIA1 and TIAL1

(A) FACS analysis of CD19$^+$ B cells in LNs from *Tia1* cKO (*Tia1$^{fl/fl}$ Tial1$^{fl/+}$ Mb1^{Cre}*), *Tial1* cKO (*Tia1$^{fl/+}$ Tial1$^{fl/fl}$ Mb1^{Cre}*), double *Tia1 Tial1* cKO (*Tia1$^{fl/fl}$ Tial1$^{fl/fl}$ Mb1^{Cre}*) mice and *Rag2$^{-/-}$* mice.

(B) Gating strategy for B cell analysis in spleen.

(C) Proportion of total CD19$^+$ B cells, follicular (FO) (CD19$^+$ CD93$^-$ CD21$^+$ CD23$^+$), marginal zone (MZ) (CD19$^+$ CD93$^-$ CD21^{hi} CD23$^-$), and transitional (T1, CD19$^+$ CD93$^+$ IgM^{hi} CD23$^-$; T2, CD19$^+$ CD93$^+$ IgM^{hi} CD23$^+$; T3, CD19$^+$ CD93$^+$ IgM$^+$ CD23$^+$) B cells in *Tia1* cKO, *Tial1* cKO, and double *Tia1 Tial1* cKO mice.

(D) Number of B cells in the spleen of mice shown in (C).

Data in (A)–(D) are from two independent experiments performed with at least three mice/genotype. In (C) and (D), each point represents an individual mouse. Mann-Whitney tests in (C) and (D). See also Figure S1.

TIAL1 have over 70% amino acid sequence homology. They bind to U-rich elements of selected RNA targets controlling their splicing and translation into proteins.[9,17–19] *Tial1* knockout (KO) mice are embryonic lethal,[20] whereas 50% of *Tia1*-KO mice die within the first 3 weeks after birth.[21] Survivors have profound immunological defects linked with exacerbated production of proinflammatory cytokines, such as TNF and IL-6.[21] In mature B cells, TIA1 acts as a translational silencer of *Trp53* mRNA.[9] However, it rapidly dissociates from its RNA targets upon induction of DNA damage, allowing rapid synthesis of P53 protein. P53 limits oncogenicity of DSBs generated during VDJ recombination.[22] P53 couples sensing of somatic mutations with cell-cycle arrest and DNA damage repair by NHEJ recombination.[23] If accumulated in its active form, P53 promotes apoptosis to remove mutant progenitor B cells and tumorigenesis.[24–26] However, impaired P53 expression and/or activity leads to genomic instability and B cell transformation.[27–29] Here, we show that TIA1 and TIAL1 not only limit the expression of P53 in pro-B cells but they also modulate a global RNA splicing program required for the expression of genes involved in DNA damage sensing and repair in pro-B cells.

RESULTS

TIA1 and TIAL1 are essential for B cell development

To explore the importance of post-transcriptional regulation by the RBPs TIA1 and TIAL1 during B cell development, we crossed *Tia1$^{fl/fl}$* and *Tial1$^{fl/fl}$* mice (Figure S1A) with *CD79a-Cre* mice (hereafter named as *Mb1^{Cre}* mice). Analysis of conditional Cre-mediated recombination of *Tia1* and *Tial1* revealed efficient gene deletion from the pro-B cell stage, as reported previously for *Mb1^{Cre}* mice[30] (Figure S1B). Further analysis of TIA1 and TIAL1 protein expression in splenic CD19$^+$ B cells from single B cell conditional KO (*Tia1* cKO or *Tial1* cKO) mice validated efficient gene deletion (Figure S1C). Therefore, these models allow us to study the intrinsic role of TIA1 and TIAL1 in B cells.

Phenotypic characterization of single *Tia1* cKO (*Tia1$^{fl/fl}$ Mb1^{Cre}*) and single *Tial1* cKO (*Tial1$^{fl/fl}$ Mb1^{Cre}*) mice showed no major differences in the percentage and number of B cells found in secondary lymphoid organs compared with control littermates (Cre-negative mice). CD19$^+$ B cells were found in similar proportions and numbers in lymph nodes (LNs) from control or single cKO mice (Figure 1A). Similarly, analysis of transitional (T), follicular (FO), and marginal zone (MZ) B cells in the spleen of single *Tia1* cKO mice showed no differences compared with control mice (Figures 1B–1D). The percentage and number of total CD19$^+$ and FO B cells was also similar in single *Tial1* cKO mice. However, there was a mild increase in the percentage and number of MZ B cells in *Tial1* cKO mice compared with

CellPress
OPEN ACCESS

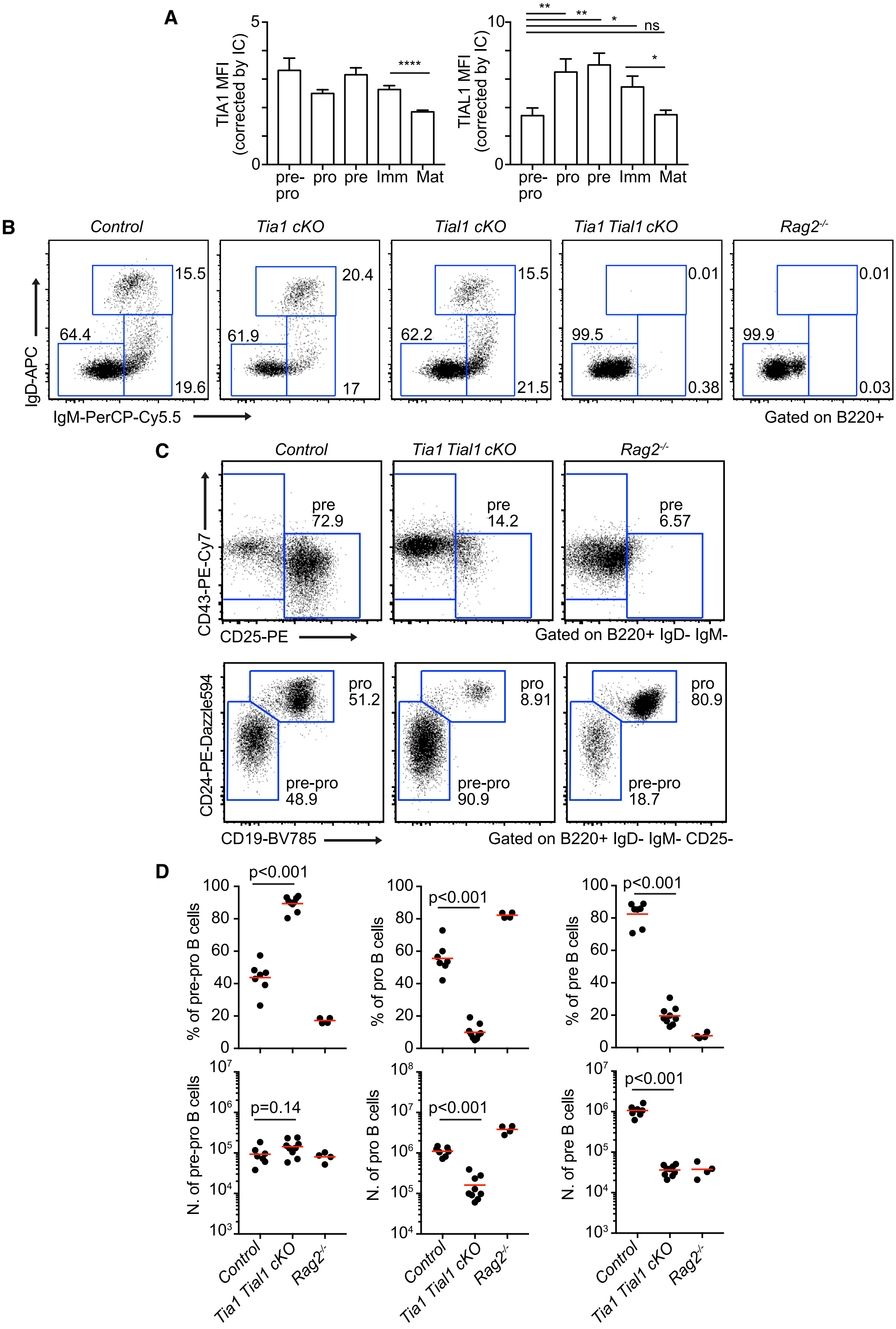

(legend on next page)

control mice. In deep contrast, double *Tia1 Tial1* cKO (*Tia1$^{fl/fl}$ Tial1$^{fl/fl}$ Mb1^{Cre}*) mice showed a profound B cell lymphopenia. CD19$^+$ B cells were absent in the LNs and spleen of these mice in a manner that resembled the phenotype of *Rag2$^{-/-}$* mice (Figure 1). Altogether, TIA1 and TIAL1 are both required for the establishment of B cells in the periphery.

To investigate whether TIA1 and TIAL1 were required for early generation of B cells in the BM, we first analyzed if the expression of these RBPs changes throughout B cell development. FACS analyses revealed no changes in TIA1 expression from early pre-pro-B cells until late immature B cells leaving the BM (Figure 2A). By contrast, TIAL1 was actively modulated during B cell development with a 2-fold increase in pro- and pre-B cells compared with pre-pro-B cell precursors (Figure 2A). The expression of TIA1 and TIAL1 was later diminished 1.5-fold in recirculating mature B cells compared with newly emerging immature BM B cells. Thus, we conclude that TIAL1, but not TIA1, is actively modulated during development of B cells.

Next, to assess the intrinsic functions of TIA1 and TIAL1 in BM B cells, we phenotyped BM B cell populations in single *Tia1* cKO and single *Tial1* cKO mice. These mice showed no defects in B cell development. The proportion and number of progenitor (B220$^+$ IgM$^-$ IgD$^-$), immature (B220$^+$ IgM$^+$ IgD$^-$), and recirculating (B220$^+$ IgM$^+$ IgD$^+$) B cells was similar in single cKO and control mice (Figure 2B). By contrast, double *Tia1 Tial1* cKO mice lacked immature and recirculating B cells (Figure 2B). Analysis of early B cell progenitors revealed a severe reduction in the percentage and number of pre-B cells in double *Tia1 Tial1* cKO mice (Figures 2C and 2D). The proportion and number of pro-B cells was also decreased by more than 5-fold in these mice compared with both control mice and *Rag2$^{-/-}$* mice. The proportion, but not the number, of pre-pro-B cells was also increased in double *Tia1 Tial1* cKO mice as a consequence of impaired B cell development at the pro-B cell stage (Figures 2C and 2D). Altogether, our results reveal that TIA1 and TIAL1 play essential, but redundant, roles during the development of B cells, and that peripheral lymphopenia is due to a severe block at the pro-B cell stage.

VDJ recombination is defective in the absence of TIA1 and TIAL1

Pro-B cells undergo programmed DNA damage and VDJ recombination of the Igμ heavy chain (*Ighm*) for later expression of a pre-BCR which drives positive cell selection and progression into the next stages of development.[31] To assess whether the block at pro-B cells found in double *Tia1 Tial1* cKO mice was associated to a defect in VDJ recombination, we quantified the presence of intracellular Igμ in BM B cells by FACS. Twenty to thirty percent of pro-B cells expressed Igμ in control mice (Figure 3A). By contrast, pro-B cells from double *Tia1 Tial1* cKO mice failed to produce Igμ. The proportion of pro-B cells found expressing Igμ in double *Tia1 Tial1* cKO mice was diminished 3-fold compared with control mice (Figures 3A and 3B). This, along with the overall reduction of the pro-B cell compartment, led to a barely detectable fraction of Igμ$^+$ pro-B cells in double cKO mice. This was comparable with the number of Igμ$^+$ pro-B cells found in *Rag2$^{-/-}$* mice (Figure 3C). In addition, the few pre-B cells found in double *Tia1 Tial1* cKO mice also failed to express Igμ (Figures 3D and 3E). This was not due to a failure in the mRNA expression of *Rag1* and *Rag2* recombinases that drive V(D)J recombination (Figure 3F). Thus, depletion of B cell progenitors in double *Tia1 Tial1* cKO mice is most likely due to a defect in IgH locus recombination and assembly of a functional pre-BCR rather than impaired *Rag-1/-2* expression.

To confirm that lack of Igμ expression was due to a failure in IgH VDJ recombination, we sorted pre-pro, pro-, and pre-B cells from control and double *Tia1 Tial1* cKO mice and assessed *Ighm locus* reorganization by qPCR.[32] Analysis of D_H-J_H and V_H-DJ_H recombination showed efficient VDJ recombination in control pro-B cells as expected (Figures 3G and 3H). However, we were unable to detect D_H-J_H recombination, and later V_H-DJ_H recombination in pro- and pre-B cells from double *Tia1 Tial1* cKO mice. No differences were found in the HS5 sequence located downstream of the IgH locus and not targeted by the recombination machinery (Figure 3H). Further analysis of Ig transcript levels in pro-B cells from control and double *Tia1 Tial1* cKO mice showed a global reduction in the expression of these genes in the absence of TIA1 and TIAL1 (Figure S2A). Altogether, TIA1 and TIAL1 are required for IgH VDJ recombination and expression of a functional pre-BCR for further progenitor cell selection and development.

TIA1 and TIAL1 preserve mRNA stability in pro-B cells

IgH VDJ recombination is tightly coupled with cell-cycle progression, chromatin replication, and DNA damage repair. Failure in any of these events leads to B cell developmental arrest. To gain mechanistic insight into how TIA1 and TIAL1 contribute to the modulation of these and other cellular processes, we performed global transcriptomics analysis in pro-B cells sorted from control and double *Tia1 Tial1* cKO mice. Principal-component analysis showed the clustering of samples per genotype, indicating that the transcriptomes of control and double cKO pro-B cells differed greatly (Figure S2D). Indeed, 1,614 genes were differentially expressed (DE) (DESeq2, FDR < 0.01) in double *Tia1 Tial1* cKO pro-B cells (Figure 4A; Table S1). A total of 690 genes was significantly increased in double *Tia1 Tial1* cKO pro-B cells, whereas 954 genes were diminished, including 121 Ig

Figure 2. TIA1 and TIAL1 are both required for B cell development

(A) TIA1 and TIAL1 protein expression measured by FACS. Mean fluorescent intensity (MFI) of TIA1 or TIAL1 was corrected by the MFI of an isotype antibody control. Data presented as mean ± SEM is from two independent experiments with n = 5 mice/experiment, unpaired t tests.

(B) FACS plots showing mature (B220$^+$ IgM$^+$ IgD$^+$), immature (B220$^+$ IgM$^+$ IgD$^-$), and precursor B cells (B220$^+$ IgM$^-$ IgD$^-$) in the BM of single *Tia1* cKO, single *Tial1* cKO, double *Tia1 Tial1* cKO, and *Rag2$^{-/-}$* mice.

(C) Gating strategy for FACS analysis of B cell precursors. Pre-B cells (B220$^+$ IgM$^-$ CD19$^+$ CD25$^+$ CD43$^-$), pro-B cells (B220$^+$ IgM$^-$ CD19$^+$ CD25$^-$ CD43$^+$ CD24$^+$), and pre-pro-B cells (B220$^+$ IgM$^-$ CD19$^-$ CD25$^-$ CD43$^+$ CD24$^+$).

(D) Percentage and number of B cell precursors in control, double *Tia1 Tial1* cKO, and *Rag2$^{-/-}$* mice.

Data in (B)–(D) are from two experiments with at least n = 4 mice/genotype. Each data point is from one mouse. Mann-Whitney tests in (D).

CellPress
OPEN ACCESS

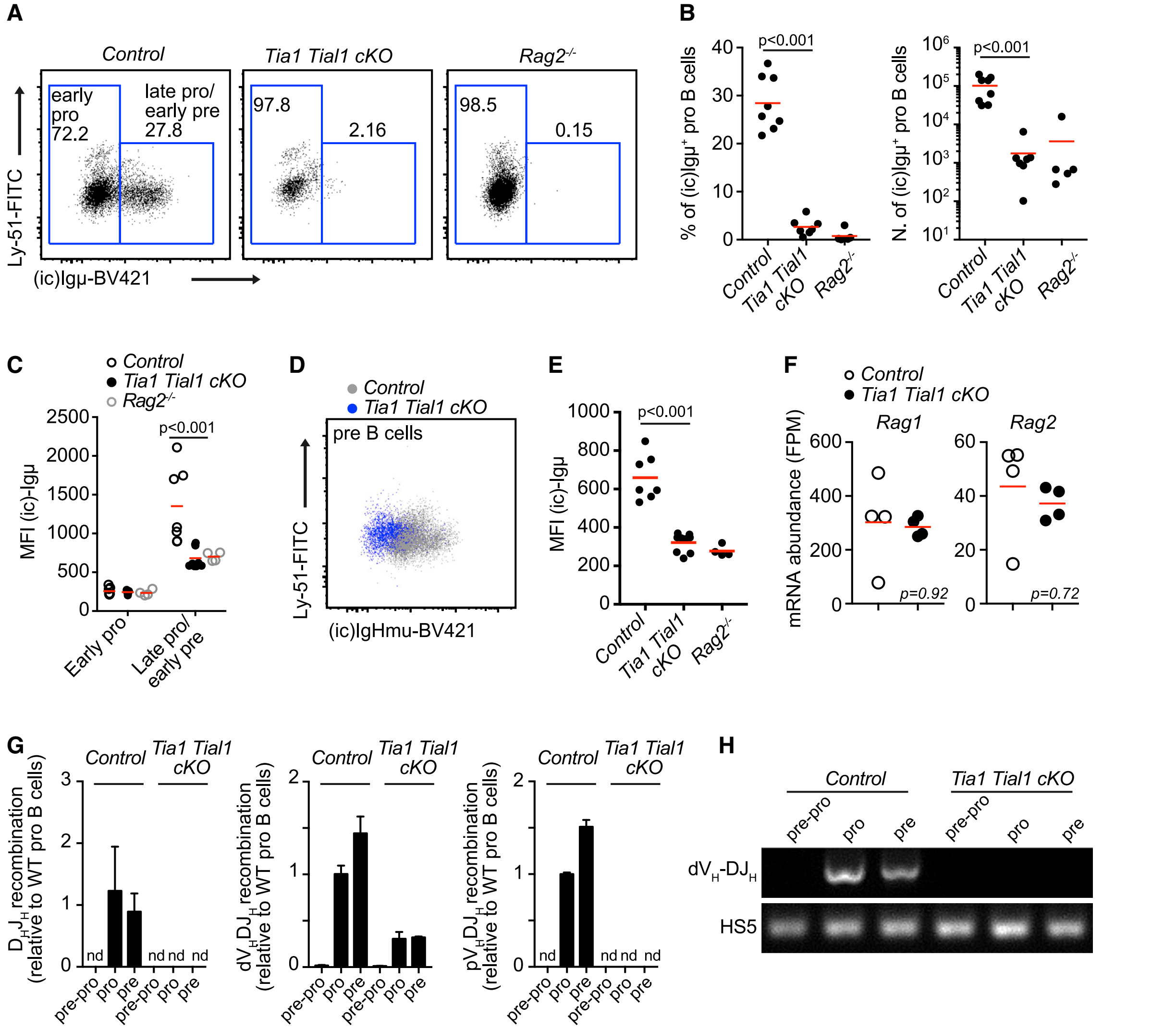

Figure 3. VDJ recombination is impaired in the absence of TIA1 and TIAL1

(A) Intracellular (ic) expression of Igμ in pro-B cells from control, double *Tia1 Tial1* cKO, and *Rag2*$^{-/-}$ mice (cells gated as B220^{+} CD19^{+} IgD^{-} IgM^{-} CD25^{-} CD43^{+} CD24^{+}, signal from *Rag2*$^{-/-}$ pro-B cells was used to set the gates for quantitation of Igμ^{+} and Igμ^{-} pro-B cells).

(B) Percentage and number of Igμ^{+} pro-B cells in mice shown in (A).

(C) MFI of Igμ in pro-B cells before and after VDJ recombination.

(D) Intracellular expression of Igμ in pre-B cells (gated as in Figure 2C).

(E) MFI of Igμ in pre-B cells.

(F) *Rag1* and *Rag2* mRNA expression in pro-B cells from control and double *Tia1 Tial1* cKO mice.

(G) Analysis by qPCR of D_H-J_H and V_H-DJ_H recombination (proximal pV_{H7183}-DJ_{H4} and distal dV_{H558}-DJ_{H2}) of the *Ighm* gene locus using genomic DNA purified from FACS-sorted pre-pro-, pro-, and pre-B cells from control and double *Tia1 Tial1* cKO mice. Data from two mice/genotype were normalized using the HS5 sequence and relative quantified to the level of VDJ recombination in control pro-B cells. Data shown as mean + SD, n.d., non-detected).

(H) Visualization of the PCR amplicons generated in the qPCR assay assessing V_H-DJ_H recombination (V_{H7183}-DJ_{H4}) in (G). HS5 genomic sequence is used as control. Representative data are shown.

Data in (A)–(E) are from two independent experiments performed with n = 3–4 mice/genotype in each experiment. Each point is data from one mouse. Mann-Whitney tests. Data in (F) are from mRNAseq, n = 4/genoptype, DESeq2 analysis, padj values calculated with BH correction of p values.

genes downregulated due to impaired VDJ recombination. Furthermore, gene set enrichment analyses (GSEA) (Table S2) revealed that genes and biological processes associated with B lymphocyte activation, signaling and differentiation were significantly decreased in double *Tia1 Tial1* cKO pro-B cells (Figures 4B and S2E). This highlights that TIA1 and TIAL1 are required for pre-BCR expression and upregulation of signal transducers for further progenitor B cell selection and development.

To understand if changes in gene expression were directly associated with RBP binding, we characterized the RNA interactome of TIAL1 in progenitor B cells expanded *in vitro*

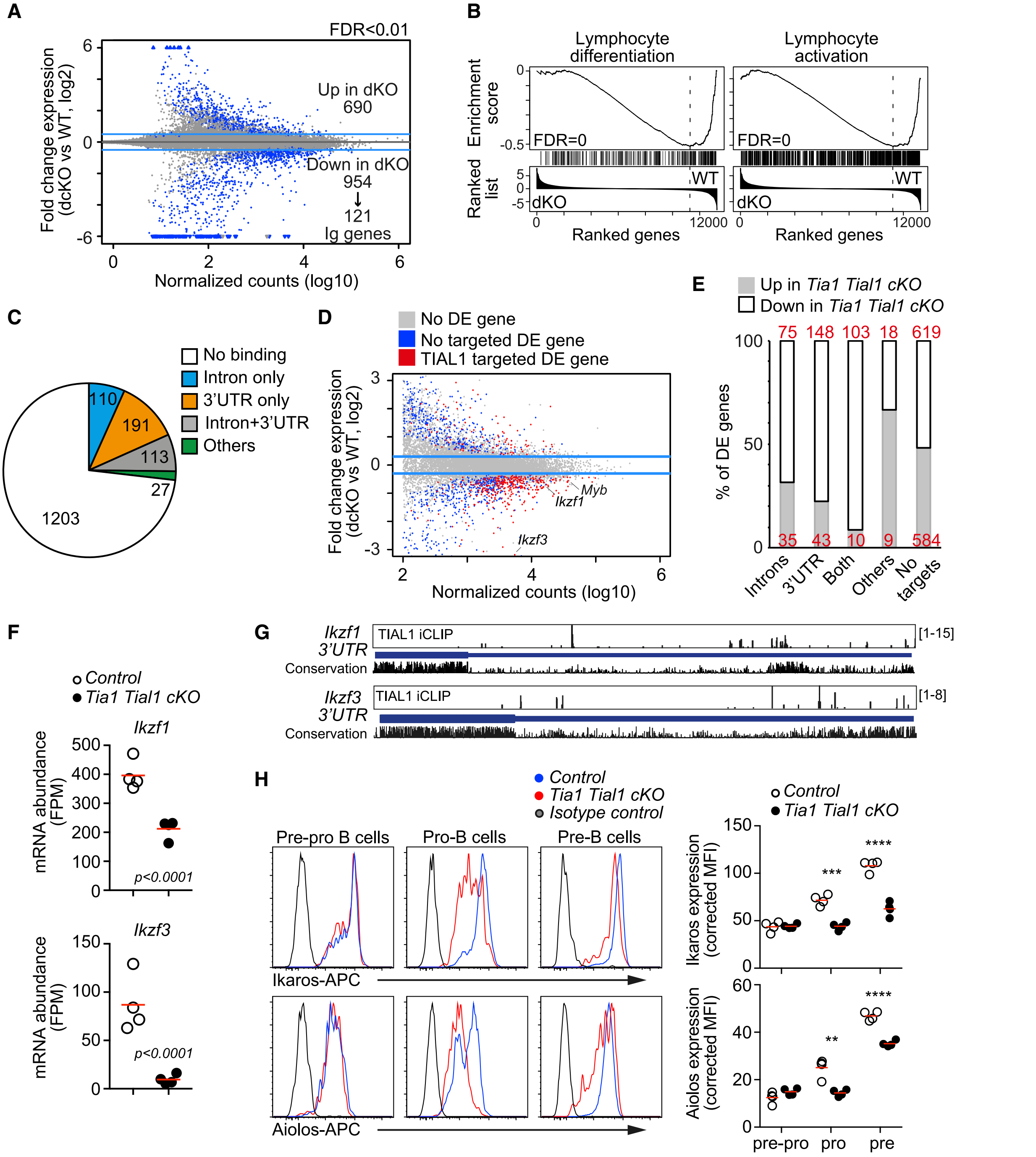

Figure 4. TIA1 and TIAL1 are required for global expression of its mRNA targets

(A) Gene expression changes in double *Tia1 Tial1* cKO pro-B cells versus control cells. Differentially expressed (DE) genes (FDR < 0.01) are in blue.

(B) Gene set enrichment analysis (GSEA) plots showing decreased expression of genes associated to lymphocyte differentiation and activation.

(C) Classification of DE genes in double *Tia1 Tial1* cKO pro-B cells based on TIAL1 binding within introns, 3′ UTRs or other genomic features (iCLIP crosslink peaks, FDR < 0.05).

(D) Changes in expression of direct RNA targets of TIAL1.

(E) Percentage of TIAL1-targeted DE genes that are up- or downregulated in double *Tia1 Tial1* cKO pro-B cells.

(F) *Ikzf1* and *Ikzf3* mRNA expression in control and double *Tia1 Tial1* cKO pro-B cells. mRNAseq data shown as fragments per million (FPM).

(G) Genomic annotation of TIAL1 crosslink sites in the last exon of *Ikzf1* and *Ikzf3*. Phylogenetic conservation in Euarchontoglires is shown (21 species, default conservation track settings from UCSC).

(legend continued on next page)

(Figure S3A).[33,34] First validation analyses of our system showed an enrichment of pro-B cells (70% of total cells) with a low proportion of cells progressing to pre-B cells (22%) or immature B cells (7%) (Figure S3B). Importantly, a small but constant proportion of pro-B cells carried out VDJ recombination and expressed intracellular Igμ (Figures S3B and S3C). This made this system suitable to capture changes in the transcriptome of developing pro-B cells. Global assessment of protein binding to RNA revealed that TIAL1 was mostly associated with U-rich elements present in introns and 3′ UTRs (Figures S3D and S3E), as shown previously in HeLa cells.[17] Transcripts from 4,126 genes were annotated as having at least one highly significant TIAL1 crosslink site (crosslink peak with FDR < 0.05). Most mRNA transcripts had TIAL1 crosslink sites annotated on both introns and 3′ UTR (Figure S3F). TIAL1 binding promoted mRNA stabilization in pro-B cells. Integration of iCLIP and transcriptomics data revealed a higher reduction in the expression of genes targeted by TIAL1 in the 3′ UTR compared with those non-targeted genes in double *Tia1 Tial1* cKO pro-B cells (Figure S3F). This reduction was milder, but still significant, for those genes only targeted by TIAL1 at intronic sequences, and it could not be detected when TIAL1 was found associated to other genomic features rather than introns and 3′ UTR. Thus, we concluded that TIAL1, and most likely TIA1, as they show a great overlap in binding properties and targets,[17] are required for the global stabilization and expression of mRNAs in pro-B cells.

Next, we assessed the direct impact of TIAL1 on driving gene expression changes in double *Tia1 Tial1* cKO pro-B cells. Four hundred and forty-one genes, of a total of 1,614 DE genes (DEseq2, FDR < 0.01), encoded transcripts targeted by TIAL1 (Figure 4C). Similar to the global trend, TIAL1 was found associated within introns and/or 3′ UTR in 94% of these genes contributing to their overall mRNA stabilization (Figure 4D). Between 70% and 91% of DE genes targeted by TIAL1 were significantly reduced in double *Tia1 Tial1* cKO pro-B cells, depending on whether TIAL1 crosslink sites were found in introns, 3′ UTR, or both (Figure 4E). Importantly, genes with which mRNA was not bound by TIAL1 were found increased or decreased in an almost similar proportion. Altogether, TIA1 and TIAL1 binding to introns and 3′ UTR preserves the expression of bound mRNAs in pro-B cells.

Among the 441 DE genes targeted by TIAL1 we found several important transcription factors that confer progenitor B cell identity and allow B cell development (Figure 4D). The mRNA expression of *Ikzf1* and *Ikzf3* was reduced 1.7- and 9.7-fold, respectively, in double *Tia1 Tial1* cKO pro-B cells compared with control cells (Figure 4F). iCLIP detected multiple TIAL1 crosslink sites in different intronic splicing sites of both *Ikzf1* and *Ikzf3* (Table S4). In addition, TIAL1 associated with U-rich elements largely conserved in euarchontoglires in the 3′ UTR of *Ikzf1* and *Ikzf3* (Figure 4G). These U-rich elements could control mRNA stability and translation into protein. Indeed, the protein expression of Ikaros and Aiolos (encoded by *Ikzf1* and *Ikzf3*, respectively) was found significantly reduced in double *Tia1 Tial1* cKO pro-B and pre-B cells (Figure 4H). In summary, our data show that post-transcriptional regulation by TIA1 and TIAL1 is required for the expression of essential transcription factors that stablish the B cell transcriptional programs for development and selection of early B cell progenitors.

TIA1 and TIAL1 are required for mRNA splicing in pro-B cells

TIA1 and TIAL1 are known splicing regulators involved in the recognition and inclusion of exons. Sequence location analysis of TIAL1 binding sites in introns confirmed previous reports indicating that TIAL1 associates to U-rich elements present in the 5′ splice site for exon definition (Figure 5A).[17–19] Therefore, TIA1 and TIAL1 could be controlling the transcriptome of pro-B cells at the qualitative level by regulating the splicing of newly synthesized RNA transcripts.

Analysis of the RNA splicing patterns in control and double *Tia1 Tial1* cKO pro-B cells using rMATS revealed thousands of alternative splicing (AS) events in the absence of TIA1 and TIAL1. A total of 3,307 AS events associated to 2,100 genes were detected in double *Tia1 Tial1* cKO pro-B cells (FDR < 0.01 and at least 10% difference in inclusion levels) (Figure 5B; Table S3). Most events corresponded to alternative spliced exons (SE) (2,015 events from 1,422 genes), but we also detected alternative events associated to retained introns (RIs) (460 events from 408 genes), mutually excluded exons (354 events from 289 genes), and alternative 5′ and 3′ splice sites (A5SS and A3SS, 248 and 230 events from 221 to 224 genes, respectively) (Figure 5B). Further analysis of the splicing patterns in control and double *Tia1 Tial1* cKO pro-B cells revealed a general loss in exon inclusion while intron retention was increased in the absence of TIA1 and TIAL1. Exon inclusion levels for 66% of the alternative SE events were reduced by more than 10% in double *Tia1 Tial1* cKO pro-B cells. By contrast, 83% of alternative RIs were included in double *Tia1 Tial1* cKO pro-B cells (Figure 5B). Taken together, our data reveal that TIA1 and TIAL1 are required for exon definition in pro-B cells and, in their absence, the splicing program in double *Tia1 Tial1* cKO pro-B cells is severely altered.

TIA1 and TIAL1 control the splicing and expression of DNA damage genes

Abnormal exon exclusion or intron inclusion can generate RNA transcripts with premature stop codons that are quickly degraded by nonsense RNA-mediated decay. Indeed, aberrant RNA splicing in double *Tia1 Tial1* cKO pro-B cells decreased the overall expression of target genes compared with control cells (Figure 5C). These AS genes were mostly associated with cellular responses to DNA damage, DNA repair, and regulation of the cell cycle as revealed by GSEA (Figure 5D; Table S2). Correlation between changes in RNA splicing and gene expression were modest with only 20% of AS genes also called as

(H) Ikaros and Aiolos protein expression analyzed by FACS. Right, MFI of Ikaros and Aiolos corrected by the MFI of an isotype antibody control. Data from one of the two independent experiments performed with n = 4 mice/genotype. Two-tailed unpaired t test (**$p < 0.01$, ***$p < 0.001$, ****$p < 0.0001$). Each point is data from one mouse. In (A), (D), and (F) mRNAseq data generated with n = 4 samples/genoptype, FDR and adjusted p values are calculated with DESeq2 using BH correction.

See also Figures S2 and S3 and Tables S1, S2, and S4.

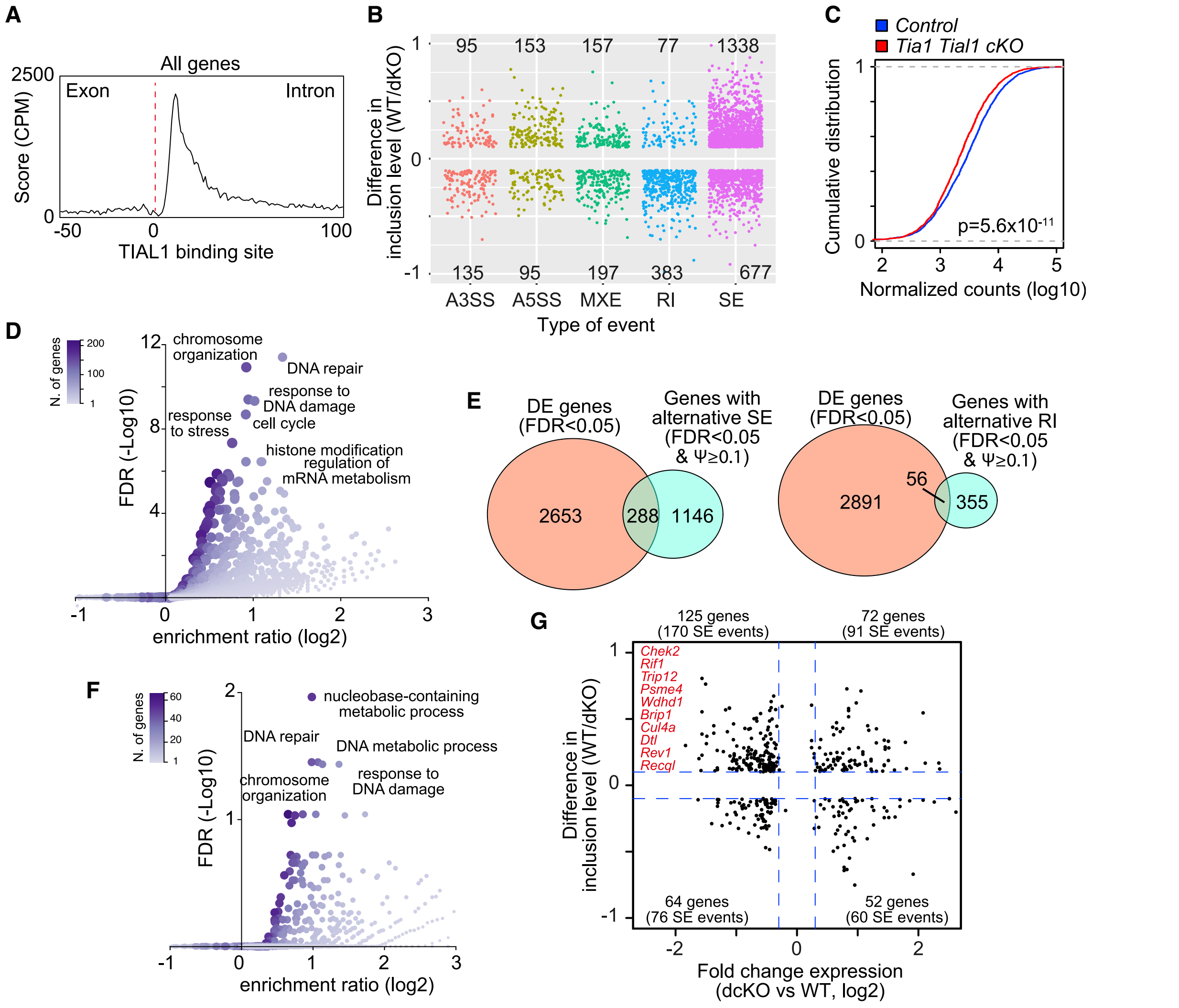

Figure 5. TIA1 and TIAL1 modulate mRNA splicing in pro-B cells

(A) Distribution of TIAL1 crosslink sites annotated within an exon-intron junction.

(B) mRNA splicing analysis in control and double *Tia1 Tial1* cKO pro-B cells using rMATS.[35] Splicing events with a difference in inclusion levels >10% and FDR < 0.01 are classified into A3SS, alternative 3′ splice site; A5SS, alternative 5′ splice site; MXE, mutually excluded exon; RI, retained intron; SE, spliced exon.

(C) Cumulative distribution analysis of the mRNA abundance of alternatively spliced (AS) genes in control and double *Tia1 Tial1* cKO pro-B cells. Kolmogorov-Smirnov test.

(D) GSEA analysis showing biological processes enriched with AS genes in double *Tia1 Tial1* cKO pro-B cells.

(E) Venn diagrams showing the correlation between AS and DE genes in double *Tia1 Tial1* cKO pro-B cells. Data divided by alternative splicing event, SE, or RI.

(F) GSEA analysis showing biological processes enriched with AS and DE genes in double *Tia1 Tial1* cKO pro-B cells.

(G) Changes in mRNA abundance and inclusion levels of AS and DE genes (FDR < 0.05, SE events with FDR < 0.01 and inclusion levels >10%). DNA damage sensing and repair genes are shown in red.

See also Figure S4 and Tables S3 and S4.

differentially expressed in double *Tia1 Tial1* cKO pro-B cells (DESeq2, FDR < 0.05) (Figure 5E). However, GSEA showed once again that these AS and DE genes were associated with DNA damage and repair (Figures 5F and 5G; Table S2). Within this gene signature, we found 24 genes required for sensing and repair of DSB, including helicases (*Setx*, *Dhx9*, and *Brip1*), ubiquitin-protein ligase (*Ubr5*, *Rfwd3*, *Trip12*, *Cul4a*, and *Dtl*), chromatin-associated protein scaffolds (*Rif1*, *Paxip1*, and *Rev1*), and signaling proteins (*Parpbp* and *Chek2*) (Figure 6A). TIAL1 was found associated to the transcripts of 22 of these 24 genes (Figure 6A; Table S4), with crosslink sites annotated preferentially to U-rich elements present in the 5′ splice site of targeted introns (Figure 6B). Further visualization of the AS events and TIAL1 crosslink sites in the gene locus of *Rif1*, a DNA damage sensor for NHEJ recombination, and *Chek2*, a signal transducer of DNA damage, confirmed the interaction of TIAL1 in the 5′ splice site downstream of the exon skipped in double *Tia1 Tial1* cKO pro-B cells (Figures 6C and S4A).

CellPress
OPEN ACCESS

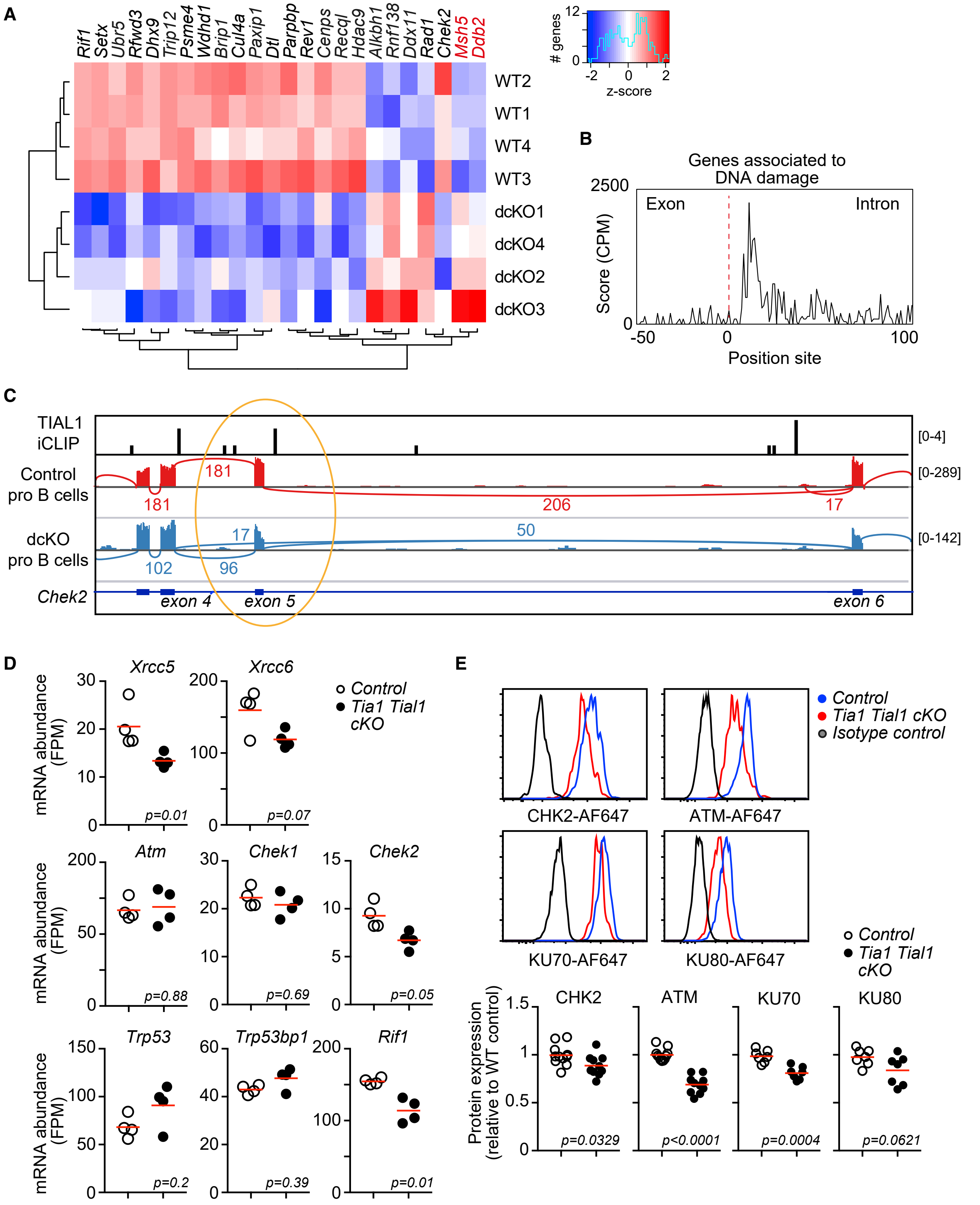

(legend on next page)

The aberrant splicing of *Rif1* and *Chek2* might promote mRNA degradation and reduced protein synthesis. Indeed, *Rif1* and *Chek2* mRNAs were significantly reduced in double *Tia1 Tial1* cKO pro-B cells compared with control cells (Figures 6A, 6D, and S4B). By contrast, there were no changes in the mRNA expression of close related genes, such as *Atm*, *Trp53*, *Chek1*, and *Trp53bp1* (Figure 6D). Quantitation of CHK2 protein abundance showed a significant reduction in double *Tia1 Tial1* cKO pro-B cells compared with control cells (Figure 6E). Further analysis of other components of the DNA damage repair machinery revealed a significant decrease in the expression of *Xrcc5* and *Xrcc6* (Figures 6D and 6E) that encode the proteins Ku70 and Ku80 required for NHEJ repair of DSB induced by Rag-1/-2.[36–39] ATM protein expression was also found significantly reduced in double *Tia1 Tial1* cKO pro-B cells, suggesting that TIA1 and TIAL1 could be also modulating mRNA translation without affecting overall mRNA abundance (Figures 6D and 6E). ATM is essential for BM development of B cells[40] and inhibition of the ATM-CHK signaling pathway induced the death of progenitor B cells *in vitro* (Figure S5). Altogether, our data demonstrate that TIA1 and TIAL1 are essential regulators of the genes sensing and repairing DNA damage in pro-B cells.

Increased DNA damage and cell death in the absence of TIA1 and TIAL1

The DNA repair machinery protects highly proliferative pro-B cells from genome instability and apoptosis.[41] Thus, we assessed the importance of TIA1 and TIAL1 during DNA damage, cell-cycle progression, and cell survival. Comet assays revealed higher genome instability in double *Tia1 Tial1* cKO pro-B cells compared with control cells (Figure 7A). Both early and late pro-B cells showed increased DNA damage in the absence of TIA1 and TIAL1, suggesting that these cells were not able to control genotoxic stress induced during VDJ recombination (Figure 7A). This correlated with higher detection of phospho-H2A.X (Ser319) (Figure 7B) and increased p53 protein levels in double *Tia1 Tial1* cKO pro-B cells (Figure 7C). As the mRNA abundance of *Trp53* was not altered in pro-B cells lacking the expression of TIA1 and TIAL1 (Figure 6D), we concluded that these RBPs could be acting in pro-B cells as silencers of *Trp53* mRNA translation as similarly shown in mature B cells.[9]

Genome instability and expression of p53 promote cell-cycle arrest and apoptosis to limit accumulation of defective pro-B cells.[26] Thus, we assessed the cell cycle in double *Tia1 Tial1* cKO pro-B cells by measuring the incorporation of the thymidine analog EdU into the genome. Lack of TIA1 and TIAL1 increased 2-fold the percentage of pro-B cells in S-phase (Figure 7D), likely reflecting a slowdown in DNA replication caused by Rif1 deficiency[42,43] and exacerbated DNA damage.[44] The percentage of double *Tia1 Tial1* cKO pro-B cells at G2/M phase also increased significantly when compared with control pro-B cells in line with previous reports.[45] In addition, analysis of active caspases with the inhibitor VAD-FMK revealed a profound increase in the percentage of apoptotic pro- and pre-B cells in double *Tia1 Tial1* cKO mice (Figures 7E and 7F). Altogether, our data demonstrate that post-transcriptional regulation by TIA1 and TIAL1 protects pro-B cells from genome instability and increased cell death.

Finally, we tested the possibility that the expression of BCR transgene could rescue B cell development in the absence of TIA1 and TIAL1 (Figure S5). To this end, we adoptively transferred BM from control *Tia1*$^{fl/fl}$ *Tial1*$^{fl/fl}$ *MD4-tg* or *Tia1*$^{fl/fl}$ *Tial1*$^{fl/fl}$ *MD4-tg Mb1*^{Cre} mice into *Rag2*$^{-/-}$ mice and assessed the different B cell populations and the DNA damage response. Overall, the number of *Tia1 Tial1* cKO MD4+ BM progenitor B cells was reduced 10-fold and they failed to reconstitute the mature B cell compartment in the periphery (Figures S5A, S5B, and S5C). pH2A.X and p53 were found significantly increased in *Tia1 Tial1* cKO MD4+ progenitor B cells compared with control MD4+ cells, in a very similar proportion to the increases reported previously in *Tia1 Tial1* cKO cells not expressing the MD4 BCR transgene (Figures S5D and S5E). In addition, changes in the cell cycle phenocopied those alterations found in *Tia1 Tial1* cKO pro-B cells (Figure S5F). Thus, we conclude that TIA1 and TIAL1 are essential for DNA damage control in pro-B cells and this is independent of the expression of a functional BCR.

DISCUSSION

The genetic programs that control B-lymphopoiesis integrate transcriptional and post-transcriptional mechanisms for timely gene expression. While the role of several transcription factors in initiating these genetic programs has now been established,[4,5] the importance of RBPs remains poorly understood. Here, we show that the RBPs TIA1 and TIAL1 are needed for genotoxic stress sensing, DNA repair, and VDJ recombination in pro-B cells. In their absence, pro-B cells fail to ensemble a functional pre-BCR leading them into apoptosis.

Our data provide evidence that TIA1 and TIAL1 play redundant functions during B cell development. TIA1 and TIAL1 share 73% of their amino acid sequence and their expression and splicing patterns can be modulated by each other.[46] This is reflected in our mouse models by the fact that TIA1 expression is increased in B cells from *Tial1 cKO* mice. Double *Tia1 Tial1 cKO* mice show

Figure 6. TIA1 and TIAL1 are required for the expression of DNA damage genes

(A) Heatmap showing the expression of AS and DE DNA damage genes in control and double *Tia1 Tial1* cKO pro-B cells. In back, TIAL1 targets.

(B) TIAL1 crosslink sites annotated within the exon-intron junctions of DNA damage genes.

(C) Alternative splicing of *Chk2* exon 5 visualized as a sashimi plot. TIAL1 crosslink sites are shown. The left scale indicates the number of unique TIAL1 iCLIP cDNA counts detected or annotated RNA-seq reads in *Chk2* exons.

(D) *Xrcc5*, *Xrcc6*, *Atm*, *Chek1*, *Chek2*, *Trp53*, *Trp53bp1*, and *Rif1a* mRNA expression in pro-B cells from control and double *Tia1 Tial1* cKO mice (mRNAseq data generated with n = 4 samples/genoptype, adjusted p values are calculated with DESeq2 using BH correction).

(E) Representative FACS histograms showing protein expression of CHK2, ATM, Ku70, and Ku80. Bottom, MFI of CHK2, ATM, Ku70, and Ku80 corrected by the MFI of an isotype antibody control. Data shown relative to the expression in control pro-B cells. Data from three independent experiments performed each with n = 3–4 mice/genotype. Each point is data from one mouse. Two-tailed unpaired t test.

See also Figure S5.

CellPress
OPEN ACCESS

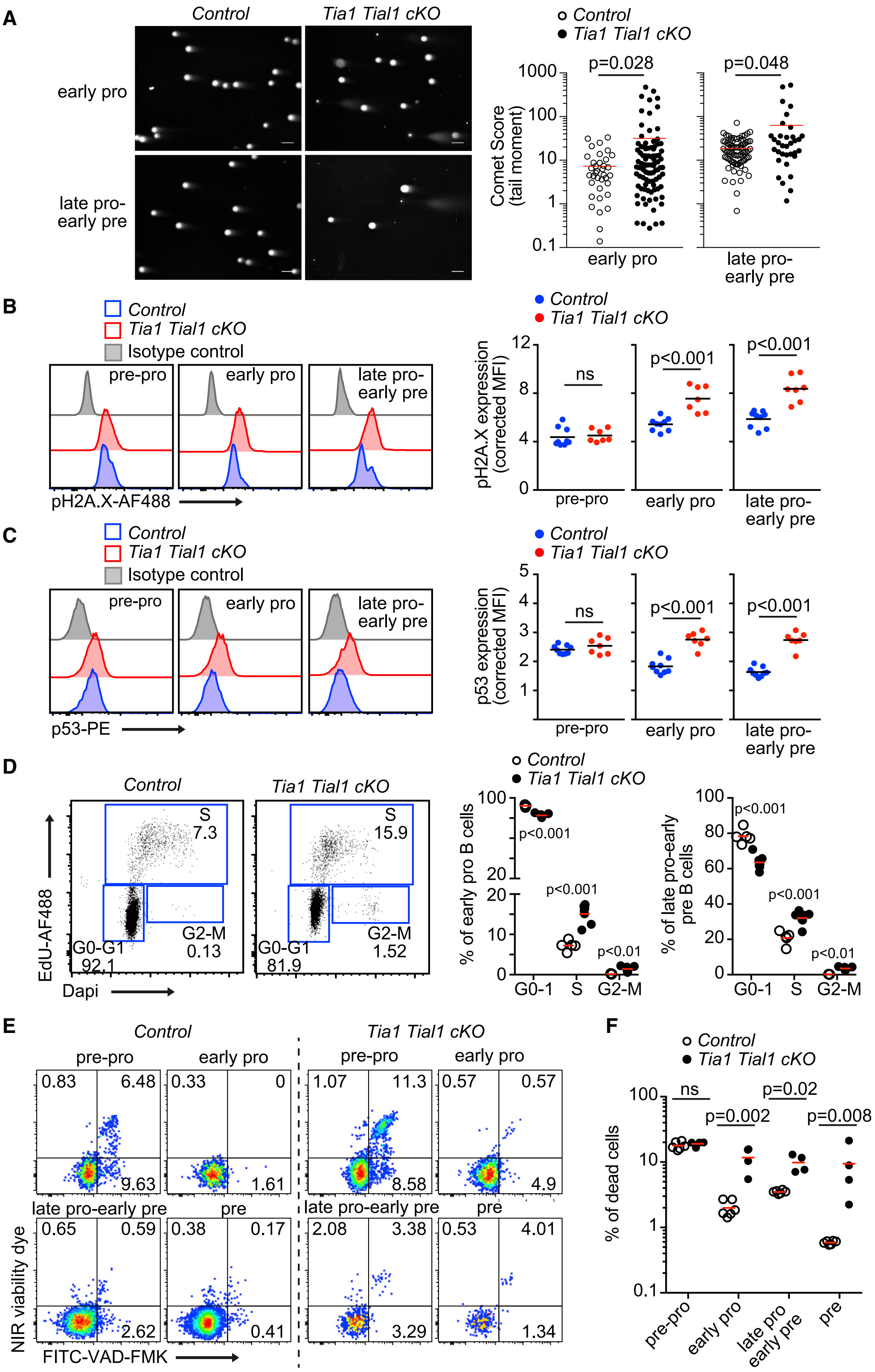

(legend on next page)

a complete developmental block at pro-B cells. By contrast, single deletion of TIA1 or TIAL1 does not impair B cell development, reinforcing the notion that TIA1 and TIAL1 are both required for B cell generation.

TIA1 and TIAL1 act as global splicing modulators in pro-B cells. Defective exon splicing and intron retention was detected in over 1,000 genes in double *Tia1 Tial1* cKO pro-B cells leading to an overall reduction in the expression of TIAL1 targeted mRNAs, likely by activating nonsense-mediated RNA decay.[47] TIA1 and TIAL1 bind at the same positions on RNAs,[17,48] where they play similar functions in exon recognition and inclusion.[49,50] Our iCLIP assays validate these findings showing the preferential binding of TIAL1 to U-rich motifs close to 5′ splice sites for exon definition.[51] This molecular mechanisms by which TIA1 and TIAL1 control AS is conserved in human and mouse, and across different cell types.

Among the many AS genes in double *Tia1 Tial1* cKO pro-B cells, we have identified several genes responsible for the sensing and repair of DNA damage, including *Chk2* and *Rif1*.[52,53] Although further investigations are required, our analyses on these genes suggest that TIA1 and TIAL1 preserve the correct splicing and expression of *Chk2* and *Rif1* rather than promoting the expression of alternative transcripts in pro-B cells. CHK2 is an essential component of the DNA damage sensing machinery that activates P53 for cell-cycle arrest and DNA repair. Across B cell development, CHK2 is required for genome stabilization,[54] whereas P53 controls B cell surveillance and, if inactivated, some B cells undergo malignant transformation.[55] Like CHK2, RIF1 is a gatekeeper for genome stability with dual functions in damage sensing and repair. On one hand, RIF1 cooperates with 53BP1 to activate NHEJ and CSR,[42] preferentially over HR.[56] On the other hand, RIF1 also acts as regulator of the cell cycle to prevent replicative stress.[52,57] In line with this, double *Tia1 Tial1* cKO pro-B cells slowly progress through S-phase and recapitulate the increase in phospho-H2A.X observed in *Rif1*$^{-/-}$ cells.[42,43] 53BP1 and RIF1 promote the recruitment of the KU70/80 complex and downstream enzymes for NHEJ repair after Rag-1/-2 activation.[36,39] The mRNA and protein expression of *Ku70* and *Ku80* is also reduced in double *Tia1 Tial1* cKO pro-B cells suggesting that a global failure in DNA damage sensing and repair is responsible for the genome instability, cell-cycle arrest, and apoptosis observed in these cells. Post-transcriptional mechanisms often coordinate the magnitude of the DNA damage response by controlling the expression and translation of specific RNA operons. Alterations in the expression of these RNA operons is often synergistic. In our model, reduced expression of KU70, KU80, RIF1, CHK2, ATM, and others, are likely responsible for the anomalous transition of double *Tia1 Tial1* cKO pro-B cells through the cell cycle. RIF1 accumulates in DNA damage foci for recruitment of KU70/80. Activation of KU70/80 enforces a G1 phase cell-cycle arrest for classical NHEJ repair of RAG-induced DNA damage.[58] Therefore, we anticipate that global reduction in the expression of these proteins in double *Tia1 Tial1* cKO pro-B cells enables transition through the S-phase for later arrest at the G2/M phase.

Comparative analyses of the splicing patterns in control and double *Tia1 Tial1* cKO pro-B cells reveal that TIA1 and TIAL1 limit intron retention, possibly by contributing to the recruitment of U1 snRNP to the 5′ splice sites during exon definition.[59,60] The need for precise exon recognition and splicing is higher in early B cell progenitors undergoing rapid proliferation and differentiation than in resting naive B cells.[61] This likely requires genome-wide modulation of the expression and function of multiple splicing factors.[61] Then, it is expected that TIA1 and TIAL1 are part of a more extended splicing network in which cooperation and competition between different RBPs shape the transcriptome of pro-B cells. Within this splicing network, it is possible that, in pro-B cells, TIA1 and TIAL1 exert antagonizing functions with other factors, such as U2AF1 and PTBP1,[62,63] which has been recently shown to be essential for cell-cycle regulation in pro-B cells.[11,64] In the future, it will be of special interest to integrate how TIA1 and TIAL1 interact with different splicing factors to further understand the contribution of RNA splicing in the dynamic regulation of the transcriptome of B cells.

The transcriptome of double *Tia1 Tial1* cKO pro-B cells plainly reflects the consequences of defective VDJ recombination and the absence of a pre-BCR. Most genes associated with B lymphocyte activation and differentiation are reduced in double *Tia1 Tial1* cKO pro-B cells likely due to a failure in positive selection. However, TIA1, and possibly TIAL1, might also contribute to B cell progenitor commitment while limiting pluripotency as suggested recently.[65] Indeed, our TIAL1:RNA interactome analyses identify several lineage-defining transcription factors as targets of TIAL1. TIAL1 binds to the 3′ UTRs of *Pax5*, *Ebf1*, *Ikzf1*, *Ikzf3*, *Myc*, and *Myb*. These genes confer B cell lineage identity and/or enable proliferation and differentiation of progenitor B cells. Double *Tia1 Tial1* cKO pro-B cells show no significant changes in the expression of *Pax5* and *Ebf1* genes compared with control cells. However, the mRNA of *Ikzf1*, *Ikzf3*, *Myc*, and *Myb* is significantly reduced in the absence TIA1 and TIAL1. Further validation showed the importance of TIA1 and TIAL1 for Ikaros and Aiolos protein synthesis (encoded by *Ikzf1* and *Ikzf3*, respectively).

Figure 7. Increase genome instability and cell dead in the absence of TIA1 and TIAL1

(A) Comet assay for analysis of double-strand DNA breaks in pro-B cells. Cells were FACS sorted prior to DNA staining with EtBr. Comet score of each cell is quantified. Data from one of the two independent experiments performed with n = 2 mice/genotype. Each point is data from one cell. Scale bar, 100 μm.

(B) Analysis of pSer139-H2A.X in B cell progenitors from control and double *Tia1 Tial1* cKO mice. MFI is shown corrected by an isotype antibody. From one of four independent experiments (n = 3–7 mice/genotype and experiment).

(C) p53 protein expression in cells shown in (B).

(D) Cell-cycle analysis in pro-B cells from control and double *Tia1 Tial1* cKO mice. Representative dot plots showing EdU incorporation and DAPI staining and the percentage of cells in each phase is shown. Quantitation from two independent experiments performed with n = 3–5 mice/genotype.

(E) FACS plots showing caspase activation and cell viability of B cell progenitors in control and double *Tia1 Tial1* cKO mice. From one of three independent experiments performed with at least n = 4 mice/genotype.

(F) Percentage of dead cells in (E).

Mann-Whitney tests in (A)–(F). In (B)–(F), each point is data from one mouse. See also Figure S6.

Mechanistically, it is possible that direct binding of TIA1 and TIAL1 within the 3′ UTR modulates mRNA translation in B cell progenitors as reported previously for *Myc* mRNA in K562 cells.[66] *Ikzf1* provides identity to pro-B cells and promotes the upregulation of signal transducers for pre-BCR signaling.[67] By contrast, *Ikzf3* limit proliferation of pre-B cells for IgL recombination.[68,69] The expression of Ikaros and Aiolos increases in pre-B cells upon signaling through the pre-BCR. Thus, the reduced expression of these proteins in pre-B cells, but not in pro-B cells, might be exacerbated due to defective VDJ recombination and pre-BCR assembly in double *Tia1 Tial1* cKO pro-B cells. Future analyses in double *Tia1 Tial1* cKO MD4-tg B cells should measure the contribution of TIA1 and TIAL1 in the expression of Ikaros and Aiolos independently of the pre-BCR.

Previously we have shown that TIA1 limits *Trp53* mRNA translation in mature B cells. After DNA damage, TIA1 dissociates from the *Trp53* 3′ UTR to enable ribosome assembly and translation into protein. This is independent of *de novo* synthesis of mRNA and feeds from the *Trp53* mRNA stored within cytoplasmic RNA granules.[9] Here, we show that, while the mRNA abundance of *Trp53* is not significantly altered in double *Tia1 Tial1* cKO pro-B cells, the protein expression of p53 is significantly increased. Although we cannot discard that this increase is not due to enhanced DNA damage and genome instability in these cells, our iCLIP assays demonstrate that TIAL1 binds to the exact U-rich regulatory elements that control TIA1-dependent translation of *Trp53* mRNA in mature B cells.

In addition to *Trp53*, it is possible that TIA1 and TIAL1 modulate translation of *Atm* and other mRNAs preventing cytotoxicity in pro-B cells. Deletion of TIA1 and TIAL1 decreases ATM protein abundance without affecting its mRNA levels. Previous studies have offered some clues about the complexity of *Atm* mRNA translation but, despite the relevance of ATM in cancer and other diseases, not much is known. *Atm* mRNA transcripts with alternative 5′ UTRs and 3″ UTRs are commonly found in lymphocytic cell lines,[70] with single-nucleotide polymorphisms being associated to chronic lymphocytic leukemia (CLL).[71] *Atm* mRNA translation is enhanced upon DNA damage induction[72] and deregulated in CLL as part of an extended post-transcriptional mechanism affecting many DNA damage sensor and repair proteins, such as ATR, RAD50, and RIF1.[73] In the future, due to the prevalence of *Atm*, *Pax5*, *Ebf1*, *Ikzf1*, *Ikzf3*, *Myc*, *Myb*, and *Trp53* mutagenesis in B cell lymphomas, it will be of special relevance to explore further how TIA1 and TIAL1 contribute to the post-transcriptional stabilization and translation of these mRNAs in both normal and cancer-transformed B cells.

TIA1 and TIAL1 are likely part of a more extended network of RBPs controlling post-transcriptional programs for dynamic modulation of gene expression at different stages of lymphocyte development. Characterization of the key RBPs in these networks and the molecular mechanisms regulated by them will be essential in the future to fully understand how transcriptional and post-transcriptional mechanisms are integrated to guarantee diversity and specificity of the adaptive immune system.

Limitations of the study

In the absence of TIA1 and TIAL1, downregulation of RIF1, KU70, KU80, ATM, CHK2, and others, has most likely a synergistic effect on how pro-B cells manage RAG1/2-mediated mutagenesis. Knockdown of these genes have profound effects on the development of B cells. However, it is likely the existence of single or combined expression thresholds for RIF1, KU70, KU80, ATM, CHK2, and others, that control cell-cycle arrest, VDJ recombination and DNA damage repair. In addition, TIA1 and TIAL1 might be relevant for the expression and/or translation of other genes involved in other aspects of the cell metabolism. The role of TIA1 and TIAL1 in mRNA translation could not be formally tested in this study due to the limited number of pro-B cells obtained to perform translation assays. Conditional deletion of TIA1 and TIAL1 in other cell types and settings will be required to identify their global versus B cell-specific functions.

STAR★METHODS

Detailed methods are provided in the online version of this paper and include the following:

- KEY RESOURCES TABLE
- RESOURCE AVAILABILITY
  - Lead contact
  - Materials availability
  - Data and code availability
- EXPERIMENTAL MODEL AND SUBJECT DETAILS
  - Primary B cell isolation and *in-vitro* culture
- METHOD DETAILS
  - Flow cytometry
  - Cell sorting by FACs
  - Analysis of VDJ recombination
  - Comet assay
  - Protein immunoblotting
  - RNA sequencing library preparation
  - Individual cross-linking immunoprecipitation (iCLIP)
  - Bioinformatics
- QUANTIFICATION AND STATISTICAL ANALYSIS

SUPPLEMENTAL INFORMATION

Supplemental information can be found online at https://doi.org/10.1016/j.celrep.2022.111869.

ACKNOWLEDGMENTS

We thank all personnel from Babraham Institute BSU, CREFRE (INSERM UMS006), INFINITy FACS, and transcriptomics facilities. M.D.D.-M. is supported by ATIP-Avenir-Plan Cancer (C18003BS), ANR (ANR-20-CE15-0007), foundation ARSEP R19201BB, foundation ARC, and La Ligue Contre Le Cancer and INSPIRE (Region Occitanie, Inserm, and CHU Toulouse). D.C.-S. is supported by Boehringer Ingelheim Fonds. M.T. is supported by a Wellcome Investigator award (200823/Z/16/Z).

AUTHOR CONTRIBUTIONS

M.D.D.-M. and I.C.O.-G. conceived, supervised, and administrated this investigation. I.C.O.-G. and M.D.D.-M. designed the methodology. I.C.O.-G., M.M., D.C.-S., and M.D.D.-M. performed, acquired, and analyzed experiments. M.D.D.-M. and Y.A. performed bioinformatics analyses. M.D.D.-M. and I.C.O.-G. interpreted the data. M.D.D.-M. and M.T. raised funding for the project. M.D.D.-M. wrote the original draft. All authors contributed with review and editing of the manuscript.

DECLARATION OF INTERESTS

The authors declare no competing interests.

INCLUSION AND DIVERSITY

We support inclusive, diverse, and equitable conduct of research.

Received: December 8, 2021
Revised: October 1, 2022
Accepted: November 29, 2022
Published: December 20, 2022

REFERENCES

1. Chi, X., Li, Y., and Qiu, X. (2020). V(D)J recombination, somatic hypermutation and class switch recombination of immunoglobulins: mechanism and regulation. Immunology *160*, 233–247. https://doi.org/10.1111/imm.13176.
2. Jung, D., Giallourakis, C., Mostoslavsky, R., and Alt, F.W. (2006). Mechanism and control of V(D)J recombination at the immunoglobulin heavy chain locus. Annu. Rev. Immunol. *24*, 541–570. https://doi.org/10.1146/annurev.immunol.23.021704.115830.
3. Bredemeyer, A.L., Helmink, B.A., Innes, C.L., Calderon, B., McGinnis, L.M., Mahowald, G.K., Gapud, E.J., Walker, L.M., Collins, J.B., Weaver, B.K., et al. (2008). DNA double-strand breaks activate a multi-functional genetic program in developing lymphocytes. Nature *456*, 819–823. https://doi.org/10.1038/nature07392.
4. Rothenberg, E.V. (2014). Transcriptional control of early T and B cell developmental choices. Annu. Rev. Immunol. *32*, 283–321. https://doi.org/10.1146/annurev-immunol-032712-100024.
5. Welinder, E., Ahsberg, J., and Sigvardsson, M. (2011). B-lymphocyte commitment: identifying the point of no return. Semin. Immunol. *23*, 335–340. https://doi.org/10.1016/j.smim.2011.08.005.
6. Diaz-Muñoz, M.D., and Osma-Garcia, I.C. (2022). The RNA regulatory programs that govern lymphocyte development and function. Wiley Interdiscip. Rev. RNA *13*, e1683. https://doi.org/10.1002/wrna.1683.
7. Dutertre, M., Lambert, S., Carreira, A., Amor-Guéret, M., and Vagner, S. (2014). DNA damage: RNA-binding proteins protect from near and far. Trends Biochem. Sci. *39*, 141–149. https://doi.org/10.1016/j.tibs.2014.01.003.
8. Reinhardt, H.C., Hasskamp, P., Schmedding, I., Morandell, S., van Vugt, M.A.T.M., Wang, X., Linding, R., Ong, S.E., Weaver, D., Carr, S.A., and Yaffe, M.B. (2010). DNA damage activates a spatially distinct late cytoplasmic cell-cycle checkpoint network controlled by MK2-mediated RNA stabilization. Mol. Cell *40*, 34–49. https://doi.org/10.1016/j.molcel.2010.09.018.
9. Díaz-Muñoz, M.D., Kiselev, V.Y., Le Novère, N., Curk, T., Ule, J., and Turner, M. (2017). Tia1 dependent regulation of mRNA subcellular location and translation controls p53 expression in B cells. Nat. Commun. *8*, 530. https://doi.org/10.1038/s41467-017-00454-2.
10. Klaric, J.A., Wüst, S., and Panier, S. (2021). New faces of old friends: emerging new roles of RNA-binding proteins in the DNA double-strand break response. Front. Mol. Biosci. *8*, 668821. https://doi.org/10.3389/fmolb.2021.668821.
11. Monzón-Casanova, E., Matheson, L.S., Tabbada, K., Zarnack, K., Smith, C.W., and Turner, M. (2020). Polypyrimidine tract-binding proteins are essential for B cell development. Elife *9*, e53557. https://doi.org/10.7554/eLife.53557.
12. Zheng, Z., Zhang, L., Cui, X.L., Yu, X., Hsu, P.J., Lyu, R., Tan, H., Mandal, M., Zhang, M., Sun, H.L., et al. (2020). Control of early B cell development by the RNA N(6)-methyladenosine methylation. Cell Rep. *31*, 107819. https://doi.org/10.1016/j.celrep.2020.107819.
13. Galloway, A., Saveliev, A., Łukasiak, S., Hodson, D.J., Bolland, D., Balmanno, K., Ahlfors, H., Monzón-Casanova, E., Mannurita, S.C., Bell, L.S., et al. (2016). RNA-binding proteins ZFP36L1 and ZFP36L2 promote cell quiescence. Science *352*, 453–459. https://doi.org/10.1126/science.aad5978.
14. Koralov, S.B., Muljo, S.A., Galler, G.R., Krek, A., Chakraborty, T., Kanellopoulou, C., Jensen, K., Cobb, B.S., Merkenschlager, M., Rajewsky, N., and Rajewsky, K. (2008). Dicer ablation affects antibody diversity and cell survival in the B lymphocyte lineage. Cell *132*, 860–874. https://doi.org/10.1016/j.cell.2008.02.020.
15. Inoue, T., Morita, M., Hijikata, A., Fukuda-Yuzawa, Y., Adachi, S., Isono, K., Ikawa, T., Kawamoto, H., Koseki, H., Natsume, T., et al. (2015). CNOT3 contributes to early B cell development by controlling Igh rearrangement and p53 mRNA stability. J. Exp. Med. *212*, 1465–1479. https://doi.org/10.1084/jem.20150384.
16. Yang, C.Y., Ramamoorthy, S., Boller, S., Rosenbaum, M., Rodriguez Gil, A., Mittler, G., Imai, Y., Kuba, K., and Grosschedl, R. (2016). Interaction of CCR4-NOT with EBF1 regulates gene-specific transcription and mRNA stability in B lymphopoiesis. Genes Dev. *30*, 2310–2324. https://doi.org/10.1101/gad.285452.116.
17. Wang, Z., Kayikci, M., Briese, M., Zarnack, K., Luscombe, N.M., Rot, G., Zupan, B., Curk, T., and Ule, J. (2010). iCLIP predicts the dual splicing effects of TIA-RNA interactions. PLoS Biol. *8*, e1000530. https://doi.org/10.1371/journal.pbio.1000530.
18. Förch, P., Puig, O., Kedersha, N., Martínez, C., Granneman, S., Séraphin, B., Anderson, P., and Valcárcel, J. (2000). The apoptosis-promoting factor TIA-1 is a regulator of alternative pre-mRNA splicing. Mol. Cell *6*, 1089–1098. https://doi.org/10.1016/s1097-2765(00)00107-6.
19. Del Gatto-Konczak, F., Bourgeois, C.F., Le Guiner, C., Kister, L., Gesnel, M.C., Stévenin, J., and Breathnach, R. (2000). The RNA-binding protein TIA-1 is a novel mammalian splicing regulator acting through intron sequences adjacent to a 5’ splice site. Mol. Cell Biol. *20*, 6287–6299. https://doi.org/10.1128/MCB.20.17.6287-6299.2000.
20. Beck, A.R., Miller, I.J., Anderson, P., and Streuli, M. (1998). RNA-binding protein TIAR is essential for primordial germ cell development. Proc. Natl. Acad. Sci. USA *95*, 2331–2336. https://doi.org/10.1073/pnas.95.5.2331.
21. Piecyk, M., Wax, S., Beck, A.R., Kedersha, N., Gupta, M., Maritim, B., Chen, S., Gueydan, C., Kruys, V., Streuli, M., and Anderson, P. (2000). TIA-1 is a translational silencer that selectively regulates the expression of TNF-alpha. EMBO J. *19*, 4154–4163. https://doi.org/10.1093/emboj/19.15.4154.
22. Guidos, C.J., Williams, C.J., Grandal, I., Knowles, G., Huang, M.T., and Danska, J.S. (1996). V(D)J recombination activates a p53-dependent DNA damage checkpoint in scid lymphocyte precursors. Genes Dev. *10*, 2038–2054. https://doi.org/10.1101/gad.10.16.2038.
23. Dujka, M.E., Puebla-Osorio, N., Tavana, O., Sang, M., and Zhu, C. (2010). ATM and p53 are essential in the cell-cycle containment of DNA breaks during V(D)J recombination in vivo. Oncogene *29*, 957–965. https://doi.org/10.1038/onc.2009.394.
24. Donehower, L.A., Harvey, M., Slagle, B.L., McArthur, M.J., Montgomery, C.A., Jr., Butel, J.S., and Bradley, A. (1992). Mice deficient for p53 are developmentally normal but susceptible to spontaneous tumours. Nature *356*, 215–221. https://doi.org/10.1038/356215a0.
25. Ventura, A., Kirsch, D.G., McLaughlin, M.E., Tuveson, D.A., Grimm, J., Lintault, L., Newman, J., Reczek, E.E., Weissleder, R., and Jacks, T. (2007). Restoration of p53 function leads to tumour regression in vivo. Nature *445*, 661–665. https://doi.org/10.1038/nature05541.
26. Slatter, T.L., Ganesan, P., Holzhauer, C., Mehta, R., Rubio, C., Williams, G., Wilson, M., Royds, J.A., Baird, M.A., and Braithwaite, A.W. (2010). p53-mediated apoptosis prevents the accumulation of progenitor B cells and B-cell tumors. Cell Death Differ. *17*, 540–550. https://doi.org/10.1038/cdd.2009.136.

27. Cheung, K.J.J., Horsman, D.E., and Gascoyne, R.D. (2009). The significance of TP53 in lymphoid malignancies: mutation prevalence, regulation, prognostic impact and potential as a therapeutic target. Br. J. Haematol. *146*, 257–269. https://doi.org/10.1111/j.1365-2141.2009.07739.x.

28. Fukasawa, K., Wiener, F., Vande Woude, G.F., and Mai, S. (1997). Genomic instability and apoptosis are frequent in p53 deficient young mice. Oncogene *15*, 1295–1302. https://doi.org/10.1038/sj.onc.1201482.

29. Zhu, C., Mills, K.D., Ferguson, D.O., Lee, C., Manis, J., Fleming, J., Gao, Y., Morton, C.C., and Alt, F.W. (2002). Unrepaired DNA breaks in p53-deficient cells lead to oncogenic gene amplification subsequent to translocations. Cell *109*, 811–821. https://doi.org/10.1016/s0092-8674(02)00770-5.

30. Hobeika, E., Thiemann, S., Storch, B., Jumaa, H., Nielsen, P.J., Pelanda, R., and Reth, M. (2006). Testing gene function early in the B cell lineage in mb1-cre mice. Proc. Natl. Acad. Sci. USA *103*, 13789–13794. https://doi.org/10.1073/pnas.0605944103.

31. Winkler, T.H., and Mårtensson, I.L. (2018). The role of the pre-B cell receptor in B cell development, repertoire selection, and tolerance. Front. Immunol. *9*, 2423. https://doi.org/10.3389/fimmu.2018.02423.

32. Braikia, F.Z., Chemin, G., Moutahir, M., and Khamlichi, A.A. (2014). Quantification of V(D)J recombination by real-time quantitative PCR. Immunol. Lett. *162*, 119–123. https://doi.org/10.1016/j.imlet.2014.08.002.

33. Holl, T.M., Haynes, B.F., and Kelsoe, G. (2010). Stromal cell independent B cell development in vitro: generation and recovery of autoreactive clones. J. Immunol. Methods *354*, 53–67. https://doi.org/10.1016/j.jim.2010.01.007.

34. von Muenchow, L., Tsapogas, P., Albertí-Servera, L., Capoferri, G., Doelz, M., Rolink, H., Bosco, N., Ceredig, R., and Rolink, A.G. (2017). Pro-B cells propagated in stromal cell-free cultures reconstitute functional B-cell compartments in immunodeficient mice. Eur. J. Immunol. *47*, 394–405. https://doi.org/10.1002/eji.201646638.

35. Shen, S., Park, J.W., Lu, Z.X., Lin, L., Henry, M.D., Wu, Y.N., Zhou, Q., and Xing, Y. (2014). rMATS: robust and flexible detection of differential alternative splicing from replicate RNA-Seq data. Proc. Natl. Acad. Sci. USA *111*, E5593–E5601. https://doi.org/10.1073/pnas.1419161111.

36. Nussenzweig, A., Chen, C., da Costa Soares, V., Sanchez, M., Sokol, K., Nussenzweig, M.C., and Li, G.C. (1996). Requirement for Ku80 in growth and immunoglobulin V(D)J recombination. Nature *382*, 551–555. https://doi.org/10.1038/382551a0.

37. Gu, Y., Jin, S., Gao, Y., Weaver, D.T., and Alt, F.W. (1997). Ku70-deficient embryonic stem cells have increased ionizing radiosensitivity, defective DNA end-binding activity, and inability to support V(D)J recombination. Proc. Natl. Acad. Sci. USA *94*, 8076–8081. https://doi.org/10.1073/pnas.94.15.8076.

38. Gu, Y., Seidl, K.J., Rathbun, G.A., Zhu, C., Manis, J.P., van der Stoep, N., Davidson, L., Cheng, H.L., Sekiguchi, J.M., Frank, K., et al. (1997). Growth retardation and leaky SCID phenotype of Ku70-deficient mice. Immunity *7*, 653–665. https://doi.org/10.1016/s1074-7613(00)80386-6.

39. Zhu, C., Bogue, M.A., Lim, D.S., Hasty, P., and Roth, D.B. (1996). Ku86-deficient mice exhibit severe combined immunodeficiency and defective processing of V(D)J recombination intermediates. Cell *86*, 379–389. https://doi.org/10.1016/s0092-8674(00)80111-7.

40. Xu, Y., Ashley, T., Brainerd, E.E., Bronson, R.T., Meyn, M.S., and Baltimore, D. (1996). Targeted disruption of ATM leads to growth retardation, chromosomal fragmentation during meiosis, immune defects, and thymic lymphoma. Genes Dev. *10*, 2411–2422. https://doi.org/10.1101/gad.10.19.2411.

41. Helmink, B.A., and Sleckman, B.P. (2012). The response to and repair of RAG-mediated DNA double-strand breaks. Annu. Rev. Immunol. *30*, 175–202. https://doi.org/10.1146/annurev-immunol-030409-101320.

42. Chapman, J.R., Barral, P., Vannier, J.B., Borel, V., Steger, M., Tomas-Loba, A., Sartori, A.A., Adams, I.R., Batista, F.D., and Boulton, S.J. (2013). RIF1 is essential for 53BP1-dependent nonhomologous end joining and suppression of DNA double-strand break resection. Mol. Cell *49*, 858–871. https://doi.org/10.1016/j.molcel.2013.01.002.

43. Saito, Y., Kobayashi, J., Kanemaki, M.T., and Komatsu, K. (2020). RIF1 controls replication initiation and homologous recombination repair in a radiation dose-dependent manner. J. Cell Sci. *133*, jcs240036. https://doi.org/10.1242/jcs.240036.

44. Chao, H.X., Poovey, C.E., Privette, A.A., Grant, G.D., Chao, H.Y., Cook, J.G., and Purvis, J.E. (2017). Orchestration of DNA damage checkpoint dynamics across the human cell cycle. Cell Syst. *5*, 445–459.e5. https://doi.org/10.1016/j.cels.2017.09.015.

45. Meyer, C., Garzia, A., Mazzola, M., Gerstberger, S., Molina, H., and Tuschl, T. (2018). The TIA1 RNA-binding protein family regulates EIF2AK2-mediated stress response and cell cycle progression. Mol. Cell *69*, 622–635.e6. https://doi.org/10.1016/j.molcel.2018.01.011.

46. Izquierdo, J.M., and Valcárcel, J. (2007). Two isoforms of the T-cell intracellular antigen 1 (TIA-1) splicing factor display distinct splicing regulation activities. Control of TIA-1 isoform ratio by TIA-1-related protein. J. Biol. Chem. *282*, 19410–19417. https://doi.org/10.1074/jbc.M700688200.

47. McGlincy, N.J., and Smith, C.W.J. (2008). Alternative splicing resulting in nonsense-mediated mRNA decay: what is the meaning of nonsense? Trends Biochem. Sci. *33*, 385–393. https://doi.org/10.1016/j.tibs.2008.06.001.

48. Aznarez, I., Barash, Y., Shai, O., He, D., Zielenski, J., Tsui, L.C., Parkinson, J., Frey, B.J., Rommens, J.M., and Blencowe, B.J. (2008). A systematic analysis of intronic sequences downstream of 5’ splice sites reveals a widespread role for U-rich motifs and TIA1/TIAL1 proteins in alternative splicing regulation. Genome Res. *18*, 1247–1258. https://doi.org/10.1101/gr.073155.107.

49. Förch, P., Puig, O., Martínez, C., Séraphin, B., and Valcárcel, J. (2002). The splicing regulator TIA-1 interacts with U1-C to promote U1 snRNP recruitment to 5’ splice sites. EMBO J. *21*, 6882–6892. https://doi.org/10.1093/emboj/cdf668.

50. Le Guiner, C., Lejeune, F., Galiana, D., Kister, L., Breathnach, R., Stévenin, J., and Del Gatto-Konczak, F. (2001). TIA-1 and TIAR activate splicing of alternative exons with weak 5’ splice sites followed by a U-rich stretch on their own pre-mRNAs. J. Biol. Chem. *276*, 40638–40646. https://doi.org/10.1074/jbc.M105642200.

51. Dember, L.M., Kim, N.D., Liu, K.Q., and Anderson, P. (1996). Individual RNA recognition motifs of TIA-1 and TIAR have different RNA binding specificities. J. Biol. Chem. *271*, 2783–2788. https://doi.org/10.1074/jbc.271.5.2783.

52. Watts, L.P., Natsume, T., Saito, Y., Garzon, J., Dong, Q., Boteva, L., Gilbert, N., Kanemaki, M.T., Hiraga, S.I., and Donaldson, A.D. (2020). The RIF1-long splice variant promotes G1 phase 53BP1 nuclear bodies to protect against replication stress. Elife *9*, e58020. https://doi.org/10.7554/eLife.58020.

53. Berge, E.O., Staalesen, V., Straume, A.H., Lillehaug, J.R., and Lønning, P.E. (2010). Chk2 splice variants express a dominant-negative effect on the wild-type Chk2 kinase activity. Biochim. Biophys. Acta *1803*, 386–395. https://doi.org/10.1016/j.bbamcr.2010.01.005.

54. El Ghamrasni, S., Pamidi, A., Halaby, M.J., Bohgaki, M., Cardoso, R., Li, L., Venkatesan, S., Sethu, S., Hirao, A., Mak, T.W., et al. (2011). Inactivation of chk2 and mus81 leads to impaired lymphocytes development, reduced genomic instability, and suppression of cancer. PLoS Genet. *7*, e1001385. https://doi.org/10.1371/journal.pgen.1001385.

55. Frank, K.M., Sharpless, N.E., Gao, Y., Sekiguchi, J.M., Ferguson, D.O., Zhu, C., Manis, J.P., Horner, J., DePinho, R.A., and Alt, F.W. (2000). DNA ligase IV deficiency in mice leads to defective neurogenesis and embryonic lethality via the p53 pathway. Mol. Cell *5*, 993–1002. https://doi.org/10.1016/s1097-2765(00)80264-6.

56. Zimmermann, M., Lottersberger, F., Buonomo, S.B., Sfeir, A., and de Lange, T. (2013). 53BP1 regulates DSB repair using Rif1 to control 5’ end resection. Science *339*, 700–704. https://doi.org/10.1126/science.1231573.

57. Cornacchia, D., Dileep, V., Quivy, J.P., Foti, R., Tili, F., Santarella-Mellwig, R., Antony, C., Almouzni, G., Gilbert, D.M., and Buonomo, S.B.C. (2012). Mouse Rif1 is a key regulator of the replication-timing programme in mammalian cells. EMBO J. *31*, 3678–3690. https://doi.org/10.1038/emboj.2012.214.

58. Liang, Z., Kumar, V., Le Bouteiller, M., Zurita, J., Kenrick, J., Lin, S.G., Lou, J., Hu, J., Ye, A.Y., Boboila, C., et al. (2021). Ku70 suppresses alternative end joining in G1-arrested progenitor B cells. Proc. Natl. Acad. Sci. USA *118*, e2103630118. https://doi.org/10.1073/pnas.2103630118.

59. Gesnel, M.C., Theoleyre, S., Del Gatto-Konczak, F., and Breathnach, R. (2007). Cooperative binding of TIA-1 and U1 snRNP in K-SAM exon splicing activation. Biochem. Biophys. Res. Commun. *358*, 1065–1070. https://doi.org/10.1016/j.bbrc.2007.05.050.

60. Zhu, H., Hasman, R.A., Young, K.M., Kedersha, N.L., and Lou, H. (2003). U1 snRNP-dependent function of TIAR in the regulation of alternative RNA processing of the human calcitonin/CGRP pre-mRNA. Mol. Cell Biol. *23*, 5959–5971. https://doi.org/10.1128/MCB.23.17.5959-5971.2003.

61. Ullrich, S., and Guigó, R. (2020). Dynamic changes in intron retention are tightly associated with regulation of splicing factors and proliferative activity during B-cell development. Nucleic Acids Res. *48*, 1327–1340. https://doi.org/10.1093/nar/gkz1180.

62. Zuccato, E., Buratti, E., Stuani, C., Baralle, F.E., and Pagani, F. (2004). An intronic polypyrimidine-rich element downstream of the donor site modulates cystic fibrosis transmembrane conductance regulator exon 9 alternative splicing. J. Biol. Chem. *279*, 16980–16988. https://doi.org/10.1074/jbc.M313439200.

63. Izquierdo, J.M., Majós, N., Bonnal, S., Martínez, C., Castelo, R., Guigó, R., Bilbao, D., and Valcárcel, J. (2005). Regulation of Fas alternative splicing by antagonistic effects of TIA-1 and PTB on exon definition. Mol. Cell *19*, 475–484. https://doi.org/10.1016/j.molcel.2005.06.015.

64. Kim, S.P., Srivatsan, S.N., Chavez, M., Shirai, C.L., White, B.S., Ahmed, T., Alberti, M.O., Shao, J., Nunley, R., White, L.S., et al. (2021). Mutant U2AF1-induced alternative splicing of H2afy (macroH2A1) regulates B-lymphopoiesis in mice. Cell Rep. *36*, 109626. https://doi.org/10.1016/j.celrep.2021.109626.

65. Vivori, C., Papasaikas, P., Stadhouders, R., Di Stefano, B., Rubio, A.R., Balaguer, C.B., Generoso, S., Mallol, A., Sardina, J.L., Payer, B., et al. (2021). Dynamics of alternative splicing during somatic cell reprogramming reveals functions for RNA-binding proteins CPSF3, hnRNP UL1, and TIA1. Genome Biol. *22*, 171. https://doi.org/10.1186/s13059-021-02372-5.

66. Liao, B., Hu, Y., and Brewer, G. (2007). Competitive binding of AUF1 and TIAR to MYC mRNA controls its translation. Nat. Struct. Mol. Biol. *14*, 511–518. https://doi.org/10.1038/nsmb1249.

67. Schwickert, T.A., Tagoh, H., Gültekin, S., Dakic, A., Axelsson, E., Minnich, M., Ebert, A., Werner, B., Roth, M., Cimmino, L., et al. (2014). Stage-specific control of early B cell development by the transcription factor Ikaros. Nat. Immunol. *15*, 283–293. https://doi.org/10.1038/ni.2828.

68. Morgan, B., Sun, L., Avitahl, N., Andrikopoulos, K., Ikeda, T., Gonzales, E., Wu, P., Neben, S., and Georgopoulos, K. (1997). Aiolos, a lymphoid restricted transcription factor that interacts with Ikaros to regulate lymphocyte differentiation. EMBO J. *16*, 2004–2013. https://doi.org/10.1093/emboj/16.8.2004.

69. Thompson, E.C., Cobb, B.S., Sabbattini, P., Meixlsperger, S., Parelho, V., Liberg, D., Taylor, B., Dillon, N., Georgopoulos, K., Jumaa, H., et al. (2007). Ikaros DNA-binding proteins as integral components of B cell developmental-stage-specific regulatory circuits. Immunity *26*, 335–344. https://doi.org/10.1016/j.immuni.2007.02.010.

70. Savitsky, K., Platzer, M., Uziel, T., Gilad, S., Sartiel, A., Rosenthal, A., Elroy-Stein, O., Shiloh, Y., and Rotman, G. (1997). Ataxia-telangiectasia: structural diversity of untranslated sequences suggests complex post-transcriptional regulation of ATM gene expression. Nucleic Acids Res. *25*, 1678–1684. https://doi.org/10.1093/nar/25.9.1678.

71. Rudd, M.F., Sellick, G.S., Webb, E.L., Catovsky, D., and Houlston, R.S. (2006). Variants in the ATM-BRCA2-CHEK2 axis predispose to chronic lymphocytic leukemia. Blood *108*, 638–644. https://doi.org/10.1182/blood-2005-12-5022.

72. Powley, I.R., Kondrashov, A., Young, L.A., Dobbyn, H.C., Hill, K., Cannell, I.G., Stoneley, M., Kong, Y.W., Cotes, J.A., Smith, G.C.M., et al. (2009). Translational reprogramming following UVB irradiation is mediated by DNA-PKcs and allows selective recruitment to the polysomes of mRNAs encoding DNA repair enzymes. Genes Dev. *23*, 1207–1220. https://doi.org/10.1101/gad.516509.

73. Lezina, L., Spriggs, R.V., Beck, D., Jones, C., Dudek, K.M., Bzura, A., Jones, G.D.D., Packham, G., Willis, A.E., and Wagner, S.D. (2018). CD40L/IL-4-stimulated CLL demonstrates variation in translational regulation of DNA damage response genes including ATM. Blood Adv. *2*, 1869–1881. https://doi.org/10.1182/bloodadvances.2017015560.

74. Kranz, A., Fu, J., Duerschke, K., Weidlich, S., Naumann, R., Stewart, A.F., and Anastassiadis, K. (2010). An improved Flp deleter mouse in C57Bl/6 based on Flpo recombinase. Genesis *48*, 512–520. https://doi.org/10.1002/dvg.20641.

75. Mason, D.Y., Jones, M., and Goodnow, C.C. (1992). Development and follicular localization of tolerant B lymphocytes in lysozyme/anti-lysozyme IgM/IgD transgenic mice. Int. Immunol. *4*, 163–175. https://doi.org/10.1093/intimm/4.2.163.

76. Hao, Z., and Rajewsky, K. (2001). Homeostasis of peripheral B cells in the absence of B cell influx from the bone marrow. J. Exp. Med. *194*, 1151–1164. https://doi.org/10.1084/jem.194.8.1151.

77. Dobin, A., Davis, C.A., Schlesinger, F., Drenkow, J., Zaleski, C., Jha, S., Batut, P., Chaisson, M., and Gingeras, T.R. (2013). STAR: ultrafast universal RNA-seq aligner. Bioinformatics *29*, 15–21. https://doi.org/10.1093/bioinformatics/bts635.

78. Love, M.I., Huber, W., and Anders, S. (2014). Moderated estimation of fold change and dispersion for RNA-seq data with DESeq2. Genome Biol. *15*, 550. https://doi.org/10.1186/s13059-014-0550-8.

79. Diaz-Muñoz, M.D., Bell, S.E., Fairfax, K., Monzon-Casanova, E., Cunningham, A.F., Gonzalez-Porta, M., Andrews, S.R., Bunik, V.I., Zarnack, K., Curk, T., et al. (2015). The RNA-binding protein HuR is essential for the B cell antibody response. Nat. Immunol. *16*, 415–425. https://doi.org/10.1038/ni.3115.

80. Hallegger, M., Chakrabarti, A.M., Lee, F.C.Y., Lee, B.L., Amalietti, A.G., Odeh, H.M., Copley, K.E., Rubien, J.D., Portz, B., Kuret, K., et al. (2021). TDP-43 condensation properties specify its RNA-binding and regulatory repertoire. Cell *184*, 4680–4696.e22. https://doi.org/10.1016/j.cell.2021.07.018.

81. Zarnegar, B.J., Flynn, R.A., Shen, Y., Do, B.T., Chang, H.Y., and Khavari, P.A. (2016). irCLIP platform for efficient characterization of protein-RNA interactions. Nat. Methods *13*, 489–492. https://doi.org/10.1038/nmeth.3840.

82. Liao, Y., Wang, J., Jaehnig, E.J., Shi, Z., and Zhang, B. (2019). WebGestalt 2019: gene set analysis toolkit with revamped UIs and APIs. Nucleic Acids Res. *47*, W199–W205. https://doi.org/10.1093/nar/gkz401.

STAR★METHODS

KEY RESOURCES TABLE

REAGENT or RESOURCE	SOURCE	IDENTIFIER
Antibodies		
Anti-TIA1 antibody (clone EPR22999-80)	Abcam	Cat# ab263945; RRID: AB_2885132
Anti-TIAL1 antibody (clone D32D3)	Cell Signaling Tech	Cat# 8509; RRID: AB_10839263
Rabbit isotype antibody (clone DA1E)	Cell Signaling Tech	Cat# 3900S
Anti-mouse CD19 antibody (clone 6D5)	BioLegend	Cat# 115507; Cat# 115543; RRID: AB_313642
Anti-mouse B220 antibody (clone RA3-6B2)	BioLegend	Cat# 103206; RRID: AB_312991
Anti-mouse IgD antibody (clone 11-26.2a)	BioLegend	Cat# 405713; RRID: AB_10645480
Anti-mouse IgM antibody (clone R6-60.2)	BD Biosciences	Cat# 550881; RRID: AB_393944
Anti-mouse CD93 antibody (clone AA4.1)	BioLegend	Cat# 136509; RRID: AB_2275879
Anti-mouse CD21/35 antibody (clone 7E9)	BioLegend	Cat# 123424; RRID: AB_2629578
Anti-mouse CD23 antibody (clone B3B4)	BioLegend	Cat# 101621; RRID: AB_2563599
Anti-mouse CD25 antibody (clone PC61)	BioLegend	Cat# 102007; RRID: AB_312856
Anti-mouse CD43 antibody (clone S7)	BD Biosciences	Cat# 553269; RRID: AB_2255226
Anti-mouse CD24 antibody (clone M1/69)	BioLegend	Cat# 101838; RRID: AB_2566732
Anti-mouse Ly-51 (BP-1) antibody (clone 6C3)	BioLegend	Cat# 108305; RRID: AB_313362
Anti-mouse Igmu antibody (goat polyclonal)	Jackson Immunoresearch	Cat# 115-475-020; RRID: AB_2338789
Anti-IKAROS antibody (clone 2A9)	BioLegend	Cat# 653305; RRID: AB_2563162
Anti-AIOLOS antibody (clone 8B2)	BioLegend	Cat# 653205; RRID: AB_2563238
Anti-CHK2 antibody (rabbit polyclonal)	ThermoFisher	Cat# BS-1391R; RRID: AB_10856284
Anti-ATM antibody (clone 2C1)	Novus Biologicals	Cat# NB100-309; RRID: AB_2243346
Anti-KU70 antibody (rabbit polyclonal)	ThermoFisher	Cat# PA5-25915; RRID: AB_2543415
Anti-KU80 antibody (rabbit polyclonal)	ThermoFisher	Cat# MA5-12933; RRID: AB_10983840
Rabbit isotype antibody (clone MOPC21)	Cell Signaling Tech	Cat# 562770
Anti-H2AX (phosphoSer139) antibody (clone 2F3)	BioLegend	Cat# 613406; RRID: AB_2248011
Anti-p53 antibody (clone G59-12)	BD Biosciences	Cat# 557027; RRID: AB_396557
Chemicals, peptides, and recombinant proteins		
CaspACE™ FITC-VAD-FMK In Situ Marker	Promega	Cat# G7461
Zombie NIR Fixable Viability dye	BioLegend	Cat# BLE423106
Recombinant Murine IL-7	Peprotech	Cat# 217-17-10ug
KU55933	Sigma Aldrich	Cat# SML1109-5MG
CHK2 inhibitor II	Sigma Aldrich	Cat# C3742-5MG
AZD7762	Sigma Aldrich	Cat# SML0350-5MG
Critical commercial assays		
CometAssay Single Cell Gel Electrophoresis Assay	RD Systems	Cat# 4250-050-K
Click-iT™ Plus EdU Alexa Fluor™ 488 Flow Cytometry Assay Kit	Thermo Fisher	Cat# C10420
Foxp3 Fix/Perm Buffer Set	BioLegend	Cat# 421403
BD Cytofix/Cytoperm™ Fixation and Permeabilization Solution	BD Biosciences	Cat# 554722
DNeasy Blood & Tissue Kit	Qiagen	Cat# 69504
LightCycler® FastStart DNA Master SYBR Green I kit	Roche	Cat# 3003230001
RNeasy Micro Kit	Qiagen	Cat# 74004

(Continued on next page)

Continued		
REAGENT or RESOURCE	SOURCE	IDENTIFIER
SMARTer Stranded mRNA-seq kit	Takara	Cat# 634862
**Deposited data**		
RNAseq datasets	This paper	GEO: GSE188556
iCLIP dataset	This paper	GEO: GSE186701
**Experimental models: Organisms/strains**		
Tia1$^{fl/fl}$, *B6:Tia1*$^{tm1a\ (KOMP)Wtsi}$	KOMP-Infrafrontier	EM:14255
Tial1$^{fl/fl}$, *B6:Tia1*$^{tm1a(EUCOMM)Wtsi}$	KOMP-Infrafrontier	EM:09761
*B6:*Flpo$^{Tg(CAG-Flpo)1Afst}$	Kranz et al.[74]	The Jackson Laboratory
Mb1^{Cre}; *B6.C(Cg)-Cd79a*$^{tm1(cre)Reth/EhobJ}$	Hobeika et al.[30]	The Jackson Laboratory
MD4-tg; C57BL/6-Tg$^{(IghelMD4)4Ccg}$/J	Mason et al.[75]	The Jackson Laboratory
Rag2$^{-/-}$; B6.Cg-*Rag2*$^{tm1.1Cgn}$/J	Hao et al.[76]	The Jackson Laboratory
**Oligonucleotides**		
See Table S5		N/A
**Software and algorithms**		
R (v. 4.0.2)	https://cran.r-project.org/	N/A
Prism (v. 7.0.)	GraphPad	N/A
FASTQC-0.11.7	The Babraham Institute https://github.com/s-andrews/FastQC	N/A
STAR-2.7.5a	Dobin et al.[77]; https://github.com/alexdobin/STAR	N/A
DESeq2 (v. 1.28.1)	Love et al.[78]; https://bioconductor.org/packages/release/bioc/html/DESeq2.html	N/A
rMATS-4.0.2	Shen et al.[35]; https://rnaseq-mats.sourceforge.net/	N/A
Python-2.7.2	https://www.python.org/	N/A
CometScore 2.0	http://rexhoover.com/index.php?id=cometscore	N/A
FlowJo v10	BD Biosciences	N/A

RESOURCE AVAILABILITY

Lead contact

Further information and requests for resources and reagents should be directed to and will be fulfilled by the lead contact, Manuel D. Diaz-Munoz (manuel.diaz-munoz@inserm.fr).

Materials availability

Mouse lines used in this study are deposited in the Knockout Mouse Project (KOMP, www.komp.org, and can be ordered from Infrafrontier, EMMA [EM:14255 and EM:09761]. This study did not generate or use any other new reagents. All reagents generated in this study are available from the lead contact with a completed material transfer agreement.

Data and code availability

- Single-cell RNA-seq data have been deposited at GEO and are publicly available as of the date of publication. Accession numbers are listed in the key resources table. Original western blot images, microscope images and flow cytometry data will be shared by the lead contact upon request.
- This paper does not report original code.
- Any additional information required to reanalyse the data reported in this paper as well as the code is available from the lead contact upon request.

EXPERIMENTAL MODEL AND SUBJECT DETAILS

Knock out-first *Tia1*$^{tm1a\ (KOMP)Wtsi}$ and *Tia1*$^{tm1a(EUCOMM)Wtsi}$ mice were generated by the Welcome-Trust Sanger Institute as part of the International Knockout Mouse Consortium (IKMC) including the European Conditional Mouse Mutagenesis Program (EUCOMM, www.eucomm.org; and the Knock Out Mouse Project (KOMP, www.komp.org). These mice were crossed with Flpo$^{Tg(CAG-Flpo)1Afst}$ mice[74] to generate *Tia1*$^{tm1c(KOMP)Wtsi}$ (*Tia1*$^{fl/fl}$) and *Tia1*$^{tm1c(EUCOMM)Wtsi}$ (*Tial1*$^{fl/fl}$) mice which were subsequently bred with *Cd79a*$^{tm1(cre)Reth/EhobJ}$ (*Mb1-Cre*) mice[30] for conditional deletion of *Tia1* and *Tial1* in B cells. *C57BL/6-Tg*$^{(IghelMD4)4Ccg}$*/J* mice[75] (named here as MD4-tg mice) were from The Jackson Laboratory and crossed with *Tia1*$^{fl/fl}$ *Tial1*$^{fl/fl}$ *Mb1*^{Cre} mice. *Rag2*$^{-/-}$ mice[76] were from The Jackson Laboratory. All mice were maintained on a C57BL/6 background. Randomization, but not experimental 'blinding', was set in these studies by housing conditional KO mice and Cre-negative littermate control mice in the same cage from weaning. Male and female mice of 8–16 weeks of age were used in all experiments. No primary pathogens or additional agents listed in the FELASA recommendations were confirmed during health monitoring surveys of the mouse stock. Ambient temperature was ∼19-21C and relative humidity 52%. Lighting was provided on a 12-h light: 12-h dark cycle. After weaning, mice were transferred to individually ventilated cages with 2–5 mice per cage. Mice were fed ad libitum and received environmental enrichment. All experimental procedures were approved by the local ethical committee of INFINITy and by the French Ministry of Education, Research and Innovation.

Primary B cell isolation and *in-vitro* culture

BM B cells were isolated from the femurs and tibias of male and female mice by fluxing these bones with 5–10 mL of complete RPMI-1640 medium (Dutch Modification from Thermo Scientific) plus 10% FCS, 100 U/mL penicillin, 10 ug/mL streptomycin, 2 mM L-glutamine, 1 mM sodium pyruvate and β-mercaptoethanol (50 μM) and a 21-G needle. Single cell suspension from spleen and peripheral LNs (axillar, brachial and inguinal) was generated by smashing the tissues in complete RPMI medium with the help of a needle plunger and by passing the cells through a 40 μm-cell strainer. ACK Lysis buffer (Thermo Scientific) was used to lyse red blood cells before culturing BM cells at 37°C in a density of 0.75 × 10^6 cells/mL in IMDM medium containing Glutamax (Thermo Scientific) supplemented with 10% FBS, 50 uM β-mercaptoethanol, 100 U/mL penicillin, 10 ug/mL streptomycin and 10 ng/mL IL-7 (Peprotech).[33,34] At day 4, cells were counted and re-plated at the same density in fresh medium. If indicated, cells were treated with the ATM inhibitor KU55933, the CHK2 inhibitor II or the CHK1/2 inhibitor AZD7762, all from Sigma-Aldrich, at a dose of 1 μM. Expansion and phenotypic characterization of pro-B cells was performed at day 8 by FACs before performing downstream analyses.

METHOD DETAILS

Flow cytometry

Flow cytometry analyses of B cell populations in the BM, peripheral lymphoid organs and *in-vitro* cell cultures were performed using the antibodies indicated in Table S5. Prior cell surface marker staining, cells were incubated with the Zombie NIR Fixable Viability dye (BioLegend) and an anti-mouse Fc Receptor Blocking antibody (clone 2.4G2) resuspended in PBS+2% FCS (FACs buffer) for 15 min at 4°C. After washing, cells were incubated for 30 min at 4°C with a mix of antibodies against cell surface markers resuspended in FACs buffer. After extensive washing with FACs buffer, cells were fixed and permeabilized for 30 min at 15°C with the BD Cytofix/Cytoperm Fixation and Permeabilization Solution from BD Biosciences or with the Foxp3 Fix/Perm Buffer Set (BioLegend). Intracellular protein staining was performed in Permeabilization buffer containing 1% FCS for at least 1 h at 4°C. For assessment of cell apoptosis, BM cells were incubated with CaspACE FITC-VAD-FMK in Situ Marker (1 uM per 10^7 cells) for 30 minutes at 37°C in complete RPMI medium. Analysis of EdU incorporation was performed using the Click-iT™ Plus EdU Alexa Fluor™ 488 Flow Cytometry Assay Kit rom ThermoFisher. Briefly, 200 ul of EdU solution (2.5 mg/mL in PBS) was injected i.p. to mice 1.5 hours prior isolation of the bone marrow. After cell surface staining, cells were fixed and permeabilised for EdU detection. DAPI was added to each sample 5 minutes prior data collection. We used a BD Fortessa cytometer and analysed the data using FlowJo v10.

Cell sorting by FACs

BM B cells from *Tia1*$^{fl/fl}$ *Tial1*$^{fl/fl}$ and *Tia1*$^{fl/fl}$ *Tial1*$^{fl/fl}$ *Mb1*^{Cre} mice were FACs-sorted for transcriptomics, VDJ recombination and DNA damage analyses.[11] Briefly, IgD$^+$, IgM$^+$, CD3e$^+$, CD11b$^+$, NK1.1$^+$, Gr1$^+$ and Ter119$^+$ cells were depleted by incubating 40 × 10^6 BM cells with 3–6 μg of biotinylated antibodies against these cell surface markers resuspended in PBS +2% FCS +1 mM EDTA for 30 min on ice. After extensive washes with PBS +2% FCS +1 mM EDTA, cells were incubated with 110 μL. of anti-biotin magnetic beads for 15 min on ice and passed cells through MACs LS columns (Miltenyi). Non-labelled BM B cells were then stained with selected antibodies in PBS +2% FCS +1 mM EDTA and sorted in a BD FACSAria Fusion cell sorter (BD) as follow: Pre-pro-B cells = CD19$^-$ B220$^+$ surface IgM$^-$ CD43$^+$ CD25$^-$; Pro-B cells = CD19$^+$ B220$^+$ surface IgM$^-$ CD43$^+$ CD25$^-$ CD24$^+$ BP1$^-$; and Pre-B cells = CD19$^+$ B220$^+$ surface IgM$^-$ CD43$^-$ CD25$^+$).

Analysis of VDJ recombination

Genomic DNA (gDNA) from FACS-sorted pre-pro, pro and pre-B cells was isolated by using a DNeasy Blood & Tissue Kit from Qiagen. Quantification of genomic DNA was performed with a NanoDrop 2000 (Thermo Scientific). V(D)J recombination in the IgH locus

pV_{H7183}-DJ_{H4} and distal dV_{H558}-DJ_{H2} recombination using specific primers (Table S5) and LightCycler® FastStart DNA Master SYBR Green I kit (Roche). qPCR in a LightCycler® (Roche) was performed using the following program: incubation at 95°C for 5 min; amplification in 40 cycles of 95°C for 15 s, 60-62°C for 15 sec and 72°C for 30 s; melting curve and cooling at 10°C. ddCT analysis was performed using HS5 sequence as endogenous control and relative to V(D)J recombination in pro B cells.

Comet assay

Analysis of DNA integrity in FACs-sorted single B cell progenitors was assessed using the CometAssay reagent kit for single cell electrophoresis following the instructions from the manufactured (Trevigen). Briefly, a neutral comet assay was performed. Cells resuspended in PBS at a density of 1 × 10^5 cells/mL and mixed with molten LMAgarose (at 37°C) at a ration 1:10. 50 ul were pipetted onto CometSlide and place in the dark at 4°C for 30 min. Then, slides were immersed into Lysis Solution (1x) at 4°C, O/N. They were transferred into neutral electrophoresis buffer (1x) for 30 min before electrophoresis with a set power supply of 21 volts for 45 min at 4°C. After removal of neutral electrophoresis buffer, slides were placed in DNA precipitation solution for 30 min. at RT followed by another 30 min. incubation in 70% ethanol. Slides were let dried before DNA staining with SYBR Gold (Thermo Scientific) and imaging in an epifluorescence microscopy. Images were analysed with CometScore 2.0 using default settings.

Protein immunoblotting

Follicular B cells were isolated from the spleens of single *Tia1* cKO ($Tia1^{fl/fl}$ $Mb1^{Cre}$), single *Tial1* cKO ($Tial1^{fl/fl}$ $Mb1^{Cre}$) and Cre-negative littermate control mice with the B cell isolation kit from Miltenyi Biotec following the instructions of the manufacturer. Protein extracts were then prepared using RIPA buffer (50 mM Tris-HCl, pH 7.4, 150 mM NaCl, 1% NP-40, 0.1% SDS and 0.5% sodium deoxycholate) containing protease inhibitors (Protease inhibitor cocktail 3, Sigma Aldrich). After 15 min on ice, samples were centrifuged (17,000×g, 5 min) to recover the protein containing supernatants. Protein sample concentration was measured using a BCA protein assay (Pierce). 10% polyacrylamide-SDS gels were loaded with 20 μg. of protein lysate for gel electrophoresis. Proteins were then transferred to nitrocellulose membranes, blocked for 15 min at RT with tris-buffered saline (TBS) buffer containing 0.5% Tween-20 and 5% non-fat dry milk (blocking buffer) and immunoblotted with specific primary antibodies against Tia1, Tial1 and Hsp90 (see Table S5). Antibodies were diluted 1:1000 in blocking buffer and incubated in rotation at 4°C, O/N. After extensive washing with TBS-T, membranes were incubated with HRP-conjugated secondary antibodies diluted 1:10000 in blocking buffer for 1 h. at RT. Protein detection was performed by enhanced chemiluminescence (Amersham Pharmacia Biotech) and imaging in an Odyssey XF Imaging System (LI-COR).

RNA sequencing library preparation

For transcriptome analyses, RNA from pro-B cells (FACs-sorted as indicated above) was isolated using the RNeasy Micro Kit from Qiagen (Cat. #74004). RNA quality was analysed on the Bioanalyzer 2100 (Agilent) and quantified in a Qubit 4 Fluorometer (Thermo Scientific). 10 ng. of RNA were then used to prepare RNAseq libraries using the SMARTer Stranded mRNA-seq following the instructions of the manufacturer (TakaraBio). Four RNAseq libraries were generated per genotype from two males and two females (four biological replicates in total) using unique indexes for sample multiplexing. Libraries were then sequenced (PE, 100 bp) across two lanes on a DNBseq platform from BGI Genomics.

Individual cross-linking immunoprecipitation (iCLIP)

Analysis of the TIAL1:RNA interactome was performed by individual nucleotide cross-linking immunoprecipitation (iCLIP).[79,80] Briefly, BM cells isolated from C57BL/6 mice were cultured in the presence of 10 ng/mL IL-7 as indicated above. At day 8, cells were washed with ice-cold PBS and irradiated with UV light (600 mJ/cm2, Stratalinker 2400). RIPA buffer and sonication (10s, ×3) was used for cell lysate and clarification. After centrifugation at 15,000 rpm, gDNA was removed with TurboDNAse (ThermoFisher) and RNA was then partially digested with RNase I (0.167 U/mL, ThermoFisher) for 3 min at 37°C. Immunoprecipitation of TIAL1-RNA complexes was performed using 3 μg. of anti-TIAL1 rabbit monoclonal antibody (clone EPR11323(B), Abcam) previously coupled to protein G dynabeads (ThermoFisher) for 1 h. at RT. Rabbit mAb IgG Isotype Control (clone DA1E) was used as negative control. 3′end dephosphorilation of RNA was performed using FastAP alkaline phosphatase (ThermoFisher) and PNK (NEB) after extensive washing of the precipitates with high-salt buffer (50 mM Tris-HCl pH 7.4, 1 M NaCl, 1 mM EDTA, 1% NP-40, 0.1% SDS and 0.5% sodium deoxycholate) and with PNK washing buffer (20 mM Tris-HCl pH 7.4, 10 mM MgCl2, 0.2% Tween-20). One fourth of the sample was ligated to a pre-adenylated infra-red labelled L3-IR-App adaptor[81] using T4 RNA ligase I (NEB) and PNK. The rest of the sample was ligated to a non-labelled L3-ATT-App DNA Linker for library preparation. RNA-protein complexes were separated by SDS-Page electrophoresis, transferred to a nitrocellulose membrane and visualized in a LI-COR Odyssey system. RNA extraction was performed by incubating the nitrocellulose fragment at 50°C for 60 minutes with proteinase K in PK buffer (100 mM This-Cl pH 7.5, 100 mM NaCl, 1 mM EDTA and 0.2% SDS). RNA was isolated by phenol/clorophorm extraction and ethanol precipitation. RNA was retro transcribed into cDNA using the irCLIP_ddRT_43 primer and SuperScript IV reverse transcriptase (ThermoFisher). After cDNA purification with Agencourt AMPure XP beads (Beckman), cDNA was circularised with CircLigase II (Epicentre), amplified by PCR using Solexa P5/P7 primers and sequence in a DNBseq platform from BGI Genomics (100 bp, SE) (see Table S5 containing information about oligonucleotide sequences).

Bioinformatics

iCLIP analyses were performed using iMaps (https://imaps.goodwright.com/).[9,80] For transcriptomics analyses, reads from different sequencing lanes were concatenated using bash cat utility and quality of resulting reads was assessed with FASTQC-0.11.7 using default parameters. Then, paired-end reads were aligned to the mouse genome (GRCm38-release 102) and quantified with STAR-2.7.5a[77] (with options –runMode alignReads –outSAMtype BAM SortedByCoordinate –quantMode TranscriptomeSAM GeneCounts –twopassMode Basic, with option –sjdbOverhang 100). Alignment files were indexed using SamTools (v. 1.9).

Differential expression analyses were performed with DESeq2 (v. 1.28.1)[78] using default parameters. Conditions included genotype, sex of animals and sample preparation day to control for variation in the data due to these parameters. Changes in gene expression with a p value adjusted using Benjamini and Hochberg correction (padj) < 0.05 was considered significant. Analysis of differential alternative splicing events was performed with rMATS-4.0.2[35] and Python-2.7.2 (with options -t paired –readLength 100 –libType fr-firstrand) with the same mouse annotation used for STAR index building and read alignment. rMATS uses hierarchical modelling to calculate the inclusion levels of five types of splicing events (spliced exon, retain intron, alternative 3′UTR splice site usage; A5SS, alternative 5′UTR splice site usage and mutually excluded exon) in a given sample. Inclusion levels are denoted in rMATS as Ψ or the percentage of the exon-intron spliced in. Paired-wise comparison between control pro-B cells ($\Psi_{control}$) and double *Tia1 Tial1* cKO pro B cells (Ψ_{cKO}) was assessed to calculate differences in inclusion levels ($\Psi_{control} - \Psi_{cKO}$). Differentially spliced events in these comparisons were considered significant if the false discovery rate (FDR) was below 0.05 and have an absolute inclusion level higher than 0.1 (this is equal to 10% of the total).

Gene ontology enrichment analyses were performed using Webgestalt[82] using default settings. Selected gene ontology gene sets were from AmiGO. Data from our sequencing datasets and gene sets was extracted and plotted in R (v. 4.0.2) using ggplot2 (v3.2.1).

QUANTIFICATION AND STATISTICAL ANALYSIS

Statistics were performed in R (v. 4.0.2) or using Prism-GraphPad (v. 7.0.) software. Performed statistical tests are indicated in each figure legend. Briefly, unpaired t-tests or non-parametric Mann-Whitney tests were used for comparisons between two groups (if not stated otherwise). Kolmogorov–Smirnov test was used to assess changes in the empirical distribution function of two samples. Benjamini and Hochberg test was used for multiple testing and false discovery rate calculation.

Cell Reports, Volume *41*

Supplemental information

The splicing regulators TIA1 and TIAL1 are required for the expression of the DNA damage repair machinery during B cell lymphopoiesis

Ines C. Osma-Garcia, Dunja Capitan-Sobrino, Mailys Mouysset, Yann Aubert, Orlane Maloudi, Martin Turner, and Manuel D. Diaz-Muñoz

Suppl. Figure 1

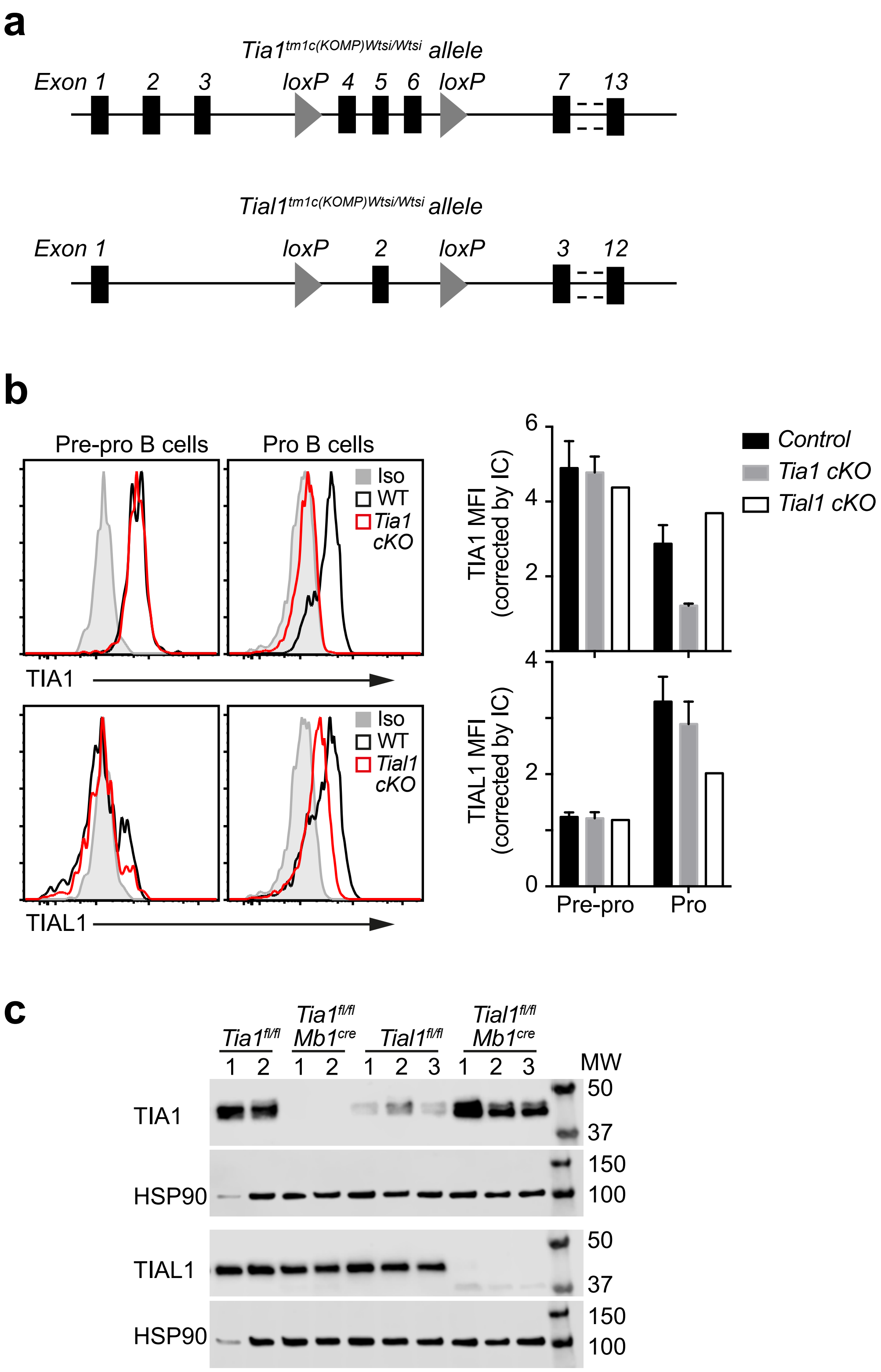

Figure S1. Targeting strategy for conditional deletion of *Tia1* and *Tial1*. Related to Figure 1.

(A) Schematic representation of the $Tia1^{tm1c(KOMP)Wtsi/Wtsi}$ and $Tial1^{tm1c(KOMP)Wtsi/Wtsi}$ alleles ($Tia1^{fl/fl}$ and $Tial1^{fl/fl}$) indicating the exons flanked by loxP sites for conditional Mb1-Cre mediated recombination.

(B) Analysis by flow cytometry of *Tia1* and *Tial1* conditional deletion in pro-B cells. Left panels, representative flow cytometry histograms showing the expression of TIA1 and TIAL1 in pre-pro B cells and pro-B cells. Right panels, quantitation of TIA1 and TIAL1 expression (mean ± SD, n=3 mice/genotype).

(C) Immunoblot of TIA1 and TIAL1 in isolated $CD19^{+}$ splenic B cells from control or single *Tia1* cKO or single *Tial1* cKO mice. HSP90 was used as loading control. Data from n=2-3 mice/genotype.

Figure S2

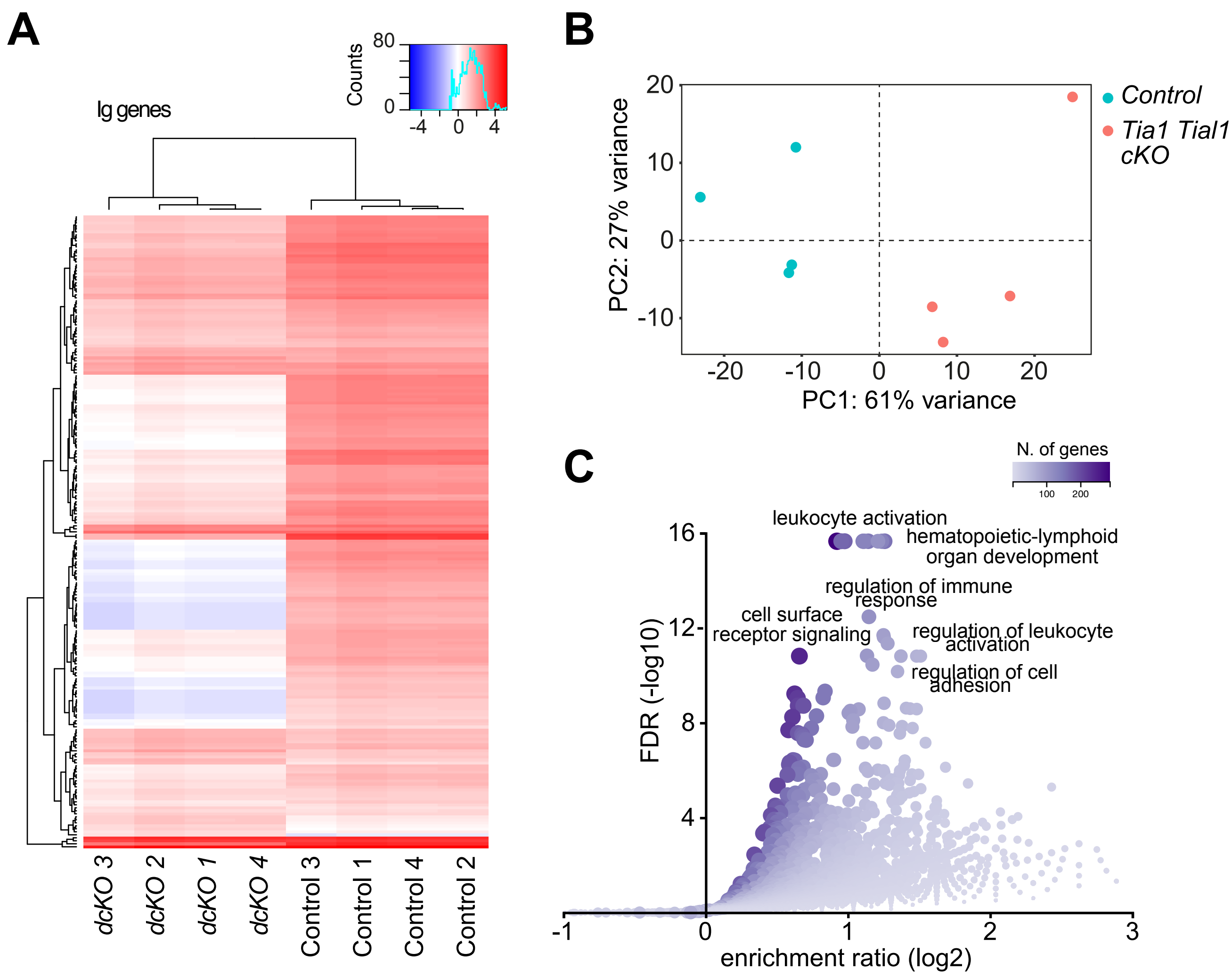
A
Ig genes
Counts
80
0
-4
0
4
dcKO 3
dcKO 2
dcKO 1
dcKO 4
Control 3
Control 1
Control 4
Control 2
B
PC2: 27% variance
PC1: 61% variance
Control
Tia1 Tial1 cKO
C
N. of genes
100
200
leukocyte activation
hematopoietic-lymphoid organ development
regulation of immune response
cell surface receptor signaling
regulation of leukocyte activation
regulation of cell adhesion
FDR (-log10)
enrichment ratio (log2)

Figure S2. Transcriptomics analysis of double *Tia1* and *Tial1* cKO pro-B cells. Related to Figure 4.

(A) Heatmap showing the expression of Ig genes in pro-B cells from control and double *Tia1* and *Tial1* cKO mice quantified by RNAseq (n=4 samples per genotype, DESeq2 analysis).

(B) Principal component analysis of transcriptomic variance in pro-B cells from control and double *Tia1* and *Tial1* cKO mice.

(C) Dot plot showing the cellular pathways that are enriched with genes differentially expressed in double *Tia1* and *Tial1 cKO* pro-B cells (GSEA performed with WebGestalt using GO terms linked to biological processes).

Figure S3

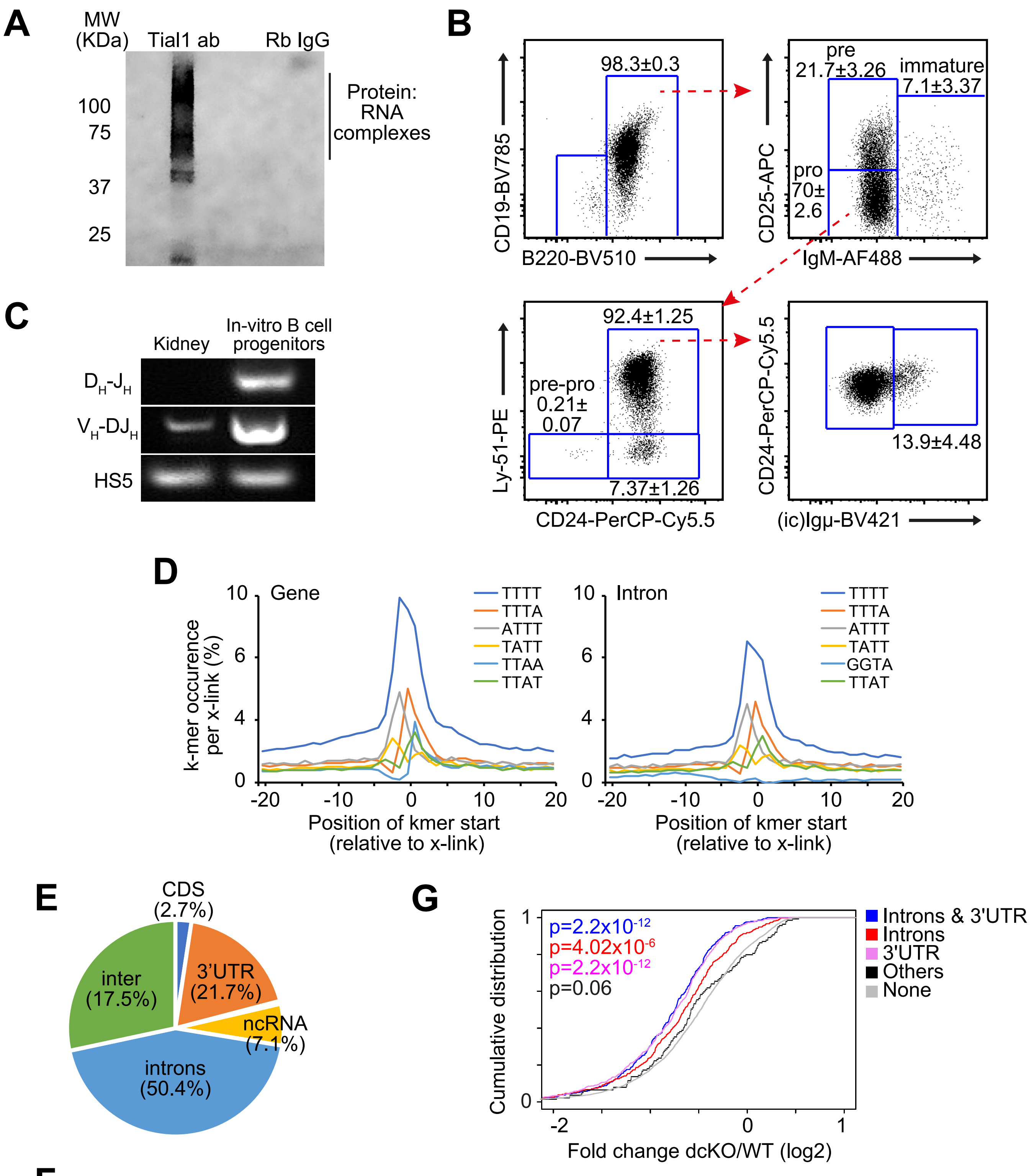

F

Xlink found in	Total genes	Genes with high confident xlink site (FDR<0.05)
Intron only	7272	913
3'UTR only	1361	1960
Intron & 3'UTR	6945	915
Others	2068	338

Figure S3. TIAL1:RNA interactome analysis in pro-B cells. Related to Figure 4.

(A) Detection of TIAL1:RNA complexes immunoprecipitated for iCLIP library construction.

(B) Characterization by flow cytometry of progenitor B cells expanded in-vitro and used for TIAL1:RNA interactome analyses.

(C) PCR analysis of VDJ recombination in *in-vitro* cultured progenitor B cells. D_H-J_H and V_H-DJ_H events were assessed after isolation of genomic DNA. HS5 sequence was used as control. Genomic DNA from the kidney of a C57BL/6 mouse was used as negative control.

(D) TIAL1 binding motif analysis. The frequency of 4-mers was quantify relative to TIAL1 cross(x)-link sites annotated to gene or intronic sequences. Only top 6 k-mer occurrences are shown.

(E) Percentage of TIAL1 crosslink sites annotated to introns, coding sequences (CDS), 3'UTRs, non-coding RNAs (ncRNAs) or intergenic sequences.

(F) Summary of target genes classified based on TIAL1 binding only in introns or 3'UTRs, or in both. The number of genes with high confident cross(x)-link sites is indicated (FDR<0.05).

(G) Cumulative distribution analysis of global changes in gene expression in double *Tia1* and *Tial1* cKO pro-B cells compared to control pro-B cells. Genes were sorted based on the detection of TIAL1 crosslink peak sites (FDR<0.05) in introns, 3'UTR and others. Kolmogorov-Smirnov tests were performed comparing a TIAL1 targeted gene group against the group of genes non-targeted by TIAL1.

Figure S4

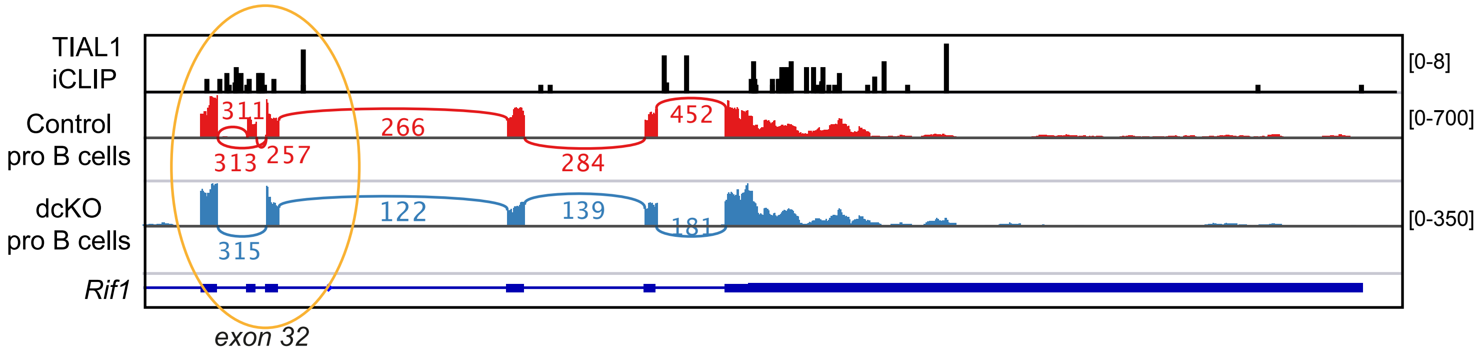

TIAL1
iCLIP
[0-8]
Control
pro B cells
[0-700]
311
266
452
313
257
284
dcKO
pro B cells
[0-350]
122
139
181
315
Rif1
exon 32

Figure S4. TIA1 and TIAL1 control *Rif1* mRNA splicing and expression. Related to Figure 5.

Sashimi plot showing alternative splicing of exon 32 of *Rif1* in pro-B cells from double *Tia1* and *Tial1* cKO mice. The number of reads annotated to the exon-exon junctions and TIAL1 crosslink sites are shown. Scales indicate the number of unique cDNA counts annotated per TIAL1 crosslink site or exon abundance.

Figure S5

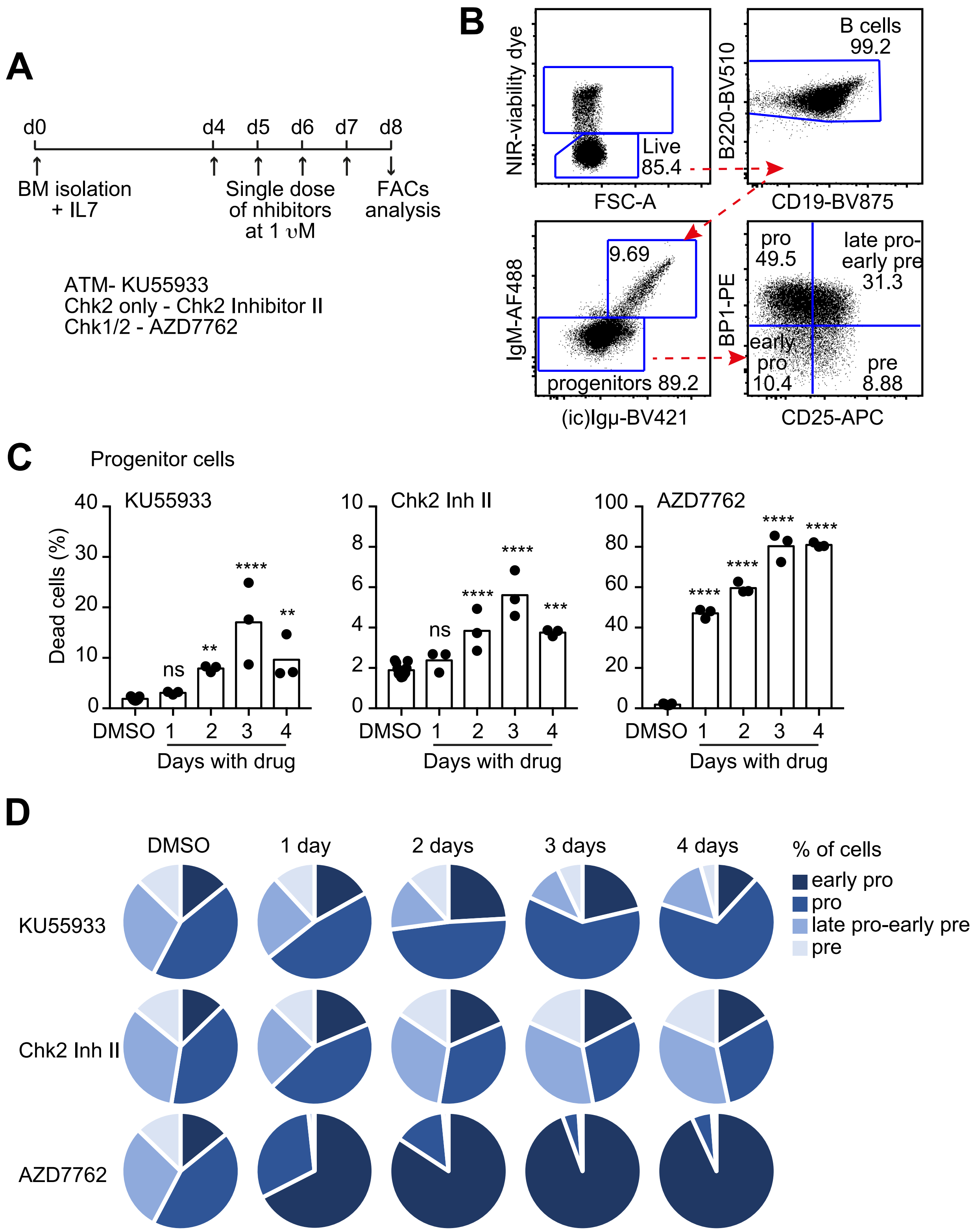

A
d0
d4
d5
d6
d7
d8
BM isolation
+ IL7
Single dose
of nhibitors
at 1 υM
FACs
analysis
ATM- KU55933
Chk2 only - Chk2 Inhibitor II
Chk1/2 - AZD7762
B
NIR-viability dye
FSC-A
Live
85.4
B220-BV510
CD19-BV875
B cells
99.2
IgM-AF488
(ic)Igμ-BV421
9.69
progenitors 89.2
BP1-PE
CD25-APC
pro
49.5
late pro-
early pre
31.3
early
pro
10.4
pre
8.88
C
Progenitor cells
Dead cells (%)
KU55933
Chk2 Inh II
AZD7762
ns
**
****
***
DMSO 1 2 3 4
Days with drug
D
DMSO
1 day
2 days
3 days
4 days
KU55933
Chk2 Inh II
AZD7762
% of cells
early pro
pro
late pro-early pre
pre

Figure S5. TIA1 and TIAL1 control *Rif1* mRNA splicing and expression. Related to Figure 6.

(A) Experimental set up to analyse the impact of ATM and CHK inhibitors in progenitor B cells *in-vitro*. Inhibitors were added into the cell culture once at the indicated time point.

(B) FACs gating strategy to assess the different subsets of progenitor B cells.

(C) Analysis of cell death in B cell cultures treated with ATM and CHK inhibitors.

(D) Pie charts showing the distribution of cell population in the cell cultures analysed in B. Data is representative from two independent experiments. In each experiment, bone marrow B cells were isolated from three mice and cultured independently to assess for biological variability. Mann-Whitney tests were performed for statistical analysis.

Figure S6

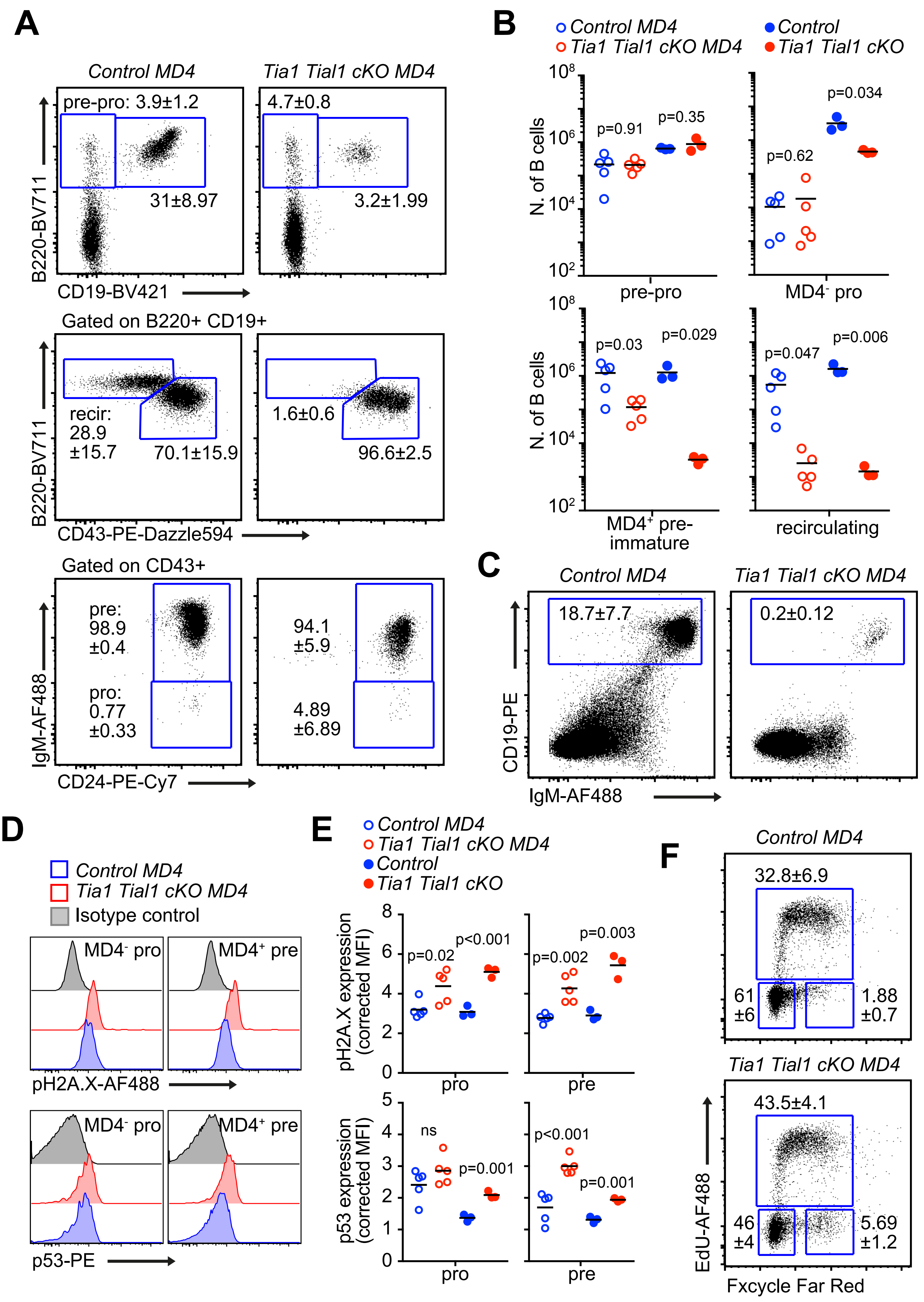
A
Control MD4
Tia1 Tial1 cKO MD4
pre-pro: 3.9±1.2
31±8.97
4.7±0.8
3.2±1.99
B220-BV711
CD19-BV421
Gated on B220+ CD19+
recir: 28.9 ±15.7
70.1±15.9
1.6±0.6
96.6±2.5
CD43-PE-Dazzle594
Gated on CD43+
pre: 98.9 ±0.4
pro: 0.77 ±0.33
94.1 ±5.9
4.89 ±6.89
IgM-AF488
CD24-PE-Cy7
B
Control MD4
Tia1 Tial1 cKO MD4
Control
Tia1 Tial1 cKO
N. of B cells
p=0.91
p=0.35
pre-pro
p=0.62
p=0.034
MD4⁻ pro
p=0.03
p=0.029
MD4⁺ pre-immature
p=0.047
p=0.006
recirculating
C
Control MD4
Tia1 Tial1 cKO MD4
18.7±7.7
0.2±0.12
CD19-PE
IgM-AF488
D
Control MD4
Tia1 Tial1 cKO MD4
Isotype control
MD4⁻ pro
MD4⁺ pre
pH2A.X-AF488
p53-PE
E
Control MD4
Tia1 Tial1 cKO MD4
Control
Tia1 Tial1 cKO
pH2A.X expression (corrected MFI)
p=0.02
p<0.001
p=0.002
p=0.003
pro
pre
p53 expression (corrected MFI)
ns
p=0.001
p<0.001
p=0.001
F
Control MD4
32.8±6.9
61 ±6
1.88 ±0.7
Tia1 Tial1 cKO MD4
43.5±4.1
46 ±4
5.69 ±1.2
EdU-AF488
Fxcycle Far Red

Figure S6. Expression of a BCR transgene fails to rescue *Tia1* and *Tial1* double KO progenitor B cells from exacerbated DNA damage. Related to Figure 7.

(A) Flow cytometry analysis of B cell subpopulations in the BM of *Rag2*$^{-/-}$ mice reconstituted with BM cells from *Tia1*$^{fl/fl}$ *Tial1*$^{fl/fl}$ MD4-tg (control MD4) mice or *Tia1*$^{fl/fl}$ *Tial1*$^{fl/fl}$ MD4-tg *Mb1*^{Cre} (*Tia1 Tial1* cKO MD4) mice. n=5 mice per genotype. The percentage of cells in each gate is shown as the mean value $\pm$ SD.

(B) Quantitation of the number of B cell progenitors in mice shown in A.

(C) Flow cytometry dot plots showing the percentage of B cells in the spleen of mice shown in A.

(D) Histograms showing the expression of pSer139-H2A.X and p53 in B cell progenitors expressing or not the MD4 BCR transgene.

(E) Quantitation of expression of pSer139-H2A.X and p53 in pro- and pre-B cells from mice described in A. Analysis of pSer139-H2A.X and p53 in *Tia1*$^{fl/fl}$ *Tial1*$^{fl/fl}$ (control) and *Tia1*$^{fl/fl}$ *Tial1*$^{fl/fl}$ *Mb1*^{Cre} (*Tia1 Tial1* cKO) was performed in parallel.

(F) Analysis of cell cycle progression in MD4^{+} progenitor B cells in mice shown in A. Unpaired t-test were performed in statistical analyses in B and E. Each data point is from one mouse.
